# Microphysiological Glomerular Filtration Barriers: Current Insights, Innovations, and Future Applications

**DOI:** 10.1002/adbi.202500108

**Published:** 2025-07-07

**Authors:** Manon Miran, Kieu Ngo, David Buob, Hanna Debiec, Pierre Ronco, Guillaume Perry

**Affiliations:** ^1^ Sorbonne Université Inserm Common and Rare Kidney Diseases: from Molecular Events to Precision Medicine CoRaKiD Paris F‐75020 France; ^2^ Sorbonne Université CNRS Laboratoire de Réactivité de Surface LRS Paris F‐75005 France; ^3^ Sorbonne Université CNRS Université Paris Saclay CentraleSupelec Laboratoire de Génie Electrique et Electronique de Paris GeePs Paris F‐75005 France

**Keywords:** glomerular filtration barrier, microfluidics, organs‐on‐a‐chip, microphysiological systems, nephrology

## Abstract

Chronic kidney disease (CKD) affects over 850 million individuals worldwide, often progressing to stages requiring dialysis or kidney transplants. Central to kidney function is the glomerular filtration barrier (GFB), which selectively filters waste while retaining essential proteins. Traditional models, including animal studies and 2D cell cultures, fail to fully replicate the GFB's complexity, limiting CKD research. Recent developments in microphysiological systems (MPS), particularly microphysiological glomerular filtration barriers (MPGFB), provide more accurate in vitro models for studying kidney diseases and evaluating therapies. MPGFB systems use organ‐on‐chip technology to integrate podocytes and glomerular endothelial cells within confined microfluidic environments, closely mimicking GFB's dynamic in vivo conditions. This setup enables detailed permeability analysis, aiding in research on disease mechanisms and drug toxicity. Furthermore, using human‐induced pluripotent stem cells in MPGFB platforms allows patient‐specific studies, enhancing insights into genetic kidney disorders. This review first examines the GFB's structure and function, focusing on its cellular and extracellular matrix components. It then discusses biological and engineering approaches to MPGFB fabrication, covering materials, 3D design, and flow control. The review concludes with MPGFB applications in disease modeling and drug testing, and addresses improvements needed for refining MPGFB as a key tool in kidney disease research and treatment.

## Graphical abstract

The glomerular filtration barrier (GFB) is the first step of blood filtration by the kidneys. The concerning increase of kidney diseases makes the development of new models essential. In this context, microphysiological glomerular filtration barriers focus on closely reproducing the physiological architecture of the in vivo GFB: podocytes, glomerular basement membrane, and endothelial cells, to reproduce its function.(Created in BioRender. Perry, G. (2025) https://BioRender.com/y90e964)

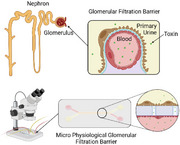

## 1 Introduction

Kidney diseases pose a significant global challenge, impacting a staggering 850 million individuals, with a particular prevalence among those from disadvantaged backgrounds.^[^ ^1^ ^]^ Chronic kidney disease (CKD), characterized by a progressive and persisting decline in renal function, ultimately necessitates dialysis or kidney transplantation in advanced stages concerning around 2 in 100 people with CKD.^[^ ^2^ ^]^ An editorial published in 2024 in Nature^[^ ^3^ ^]^ based on a review by Francis et al.^[^ ^4^ ^]^ warns about the underestimation of the dangerousness of kidney diseases, which are advancing each year worldwide and still absent from the World Health Organization list of major non‐communicable diseases causing premature death.^[^ ^5^ ^]^ CKD is a silent killer which can be hard to diagnose, as there can be little to no symptoms up to 80% of kidney function deterioration. Various factors can contribute to CKD, including underlying medical conditions such as diabetes or hypertension, drug toxicity, or even more unexpected sources, like cosmetics.^[^ ^6^ ^]^ Furthermore, the COVID‐19 crisis has had a profound impact on individuals with CKD, resulting in a COVID‐19 mortality rate that was over twice as high as that of the general population and limiting the access to diagnosis and care.^[^ ^7^ ^]^

Hence, delving into the intricate mechanisms at play becomes imperative to gain a comprehensive understanding of CKD's psychopathology and its associated consequences. In this regard, the investigation of kidney models holds great significance, offering valuable insights into the underlying processes involved and potentially pave the way for innovative therapeutic interventions. Improving the models used is a crucial aspect. Traditionally, animal models or 2D in vitro human cell culture are used but have inherent limitations that will be detailed later. A third type of model has emerged in the last decade: 3D dynamic cultures offering greater physiological relevance thanks to the development of microfluidic technology.

Microfluidics is the study and manipulation of small volumes of fluids at the micrometer scale. It offers many advantages, such as minimal sample and reagent consumption, making it more environmentally friendly and cost‐effective compared to traditional methods. Moreover, it enables easy manipulations, precise control over fluids, and high throughput screening.^[^ ^8^ ^]^ It has various applications, such as medicine, chemistry, physics, and many more. It also emerges as a powerful tool in the study of cells and organs as the shear stress plays a crucial role in tissue development and maturation. More generally, microfluidics enables better control over the cellular environment. Such devices are called Micro Physiological Systems, or MPS. The National Centre for the Replacement, Refinement, and Reduction of Animal Research in the United Kingdom defines MPS as “in vitro platforms composed of cells or tissues of human or animal origin exposed to a microenvironment designed to mimic the physiological aspects of tissue and organ function”.^[^ ^9^ ^]^ The more known term “organ‐on‐chip” represents a sub‐section of MPS. The technology has been introduced in 2010 by Huh et al.^[^ ^10^ ^]^ who reported an MPS focusing on the alveolar‐capillary interface of the lung. Since then, every organ has had one or several functions replicated on a chip. Kidneys are not an exception.

This review will focus on the glomerular filtration barrier (GFB), a key component of the kidney's functional unit, examining both its intrinsic properties and its MPS analog, the Microphysiological Glomerular Filtration Barrier (MPGFB). We will explore the physiopathological relevance of the GFB and discuss the engineering processes involved in MPGFB development, including material selection and characterization methods.

The review begins with an overview of the GFB's fundamental properties, providing essential background for readers less familiar with the subject. The second section delves into MPGFB from a biological perspective, while the third focuses on the engineering approaches underpinning their construction. The final section addresses the applications of these innovative devices, highlighting their potential in research and therapeutic contexts.

## 2 Physiology of the Glomerulus

### 2.1 Physiology

It takes about 5 min for all the blood in the body to pass through the kidneys. Every day, 180 L pass through the filtration barrier forming the primary urine. Most of it is later returned to the body, and the remains of this process form about 1.5 L of urine.^[^ ^11^ ^]^

**Figure 1:**
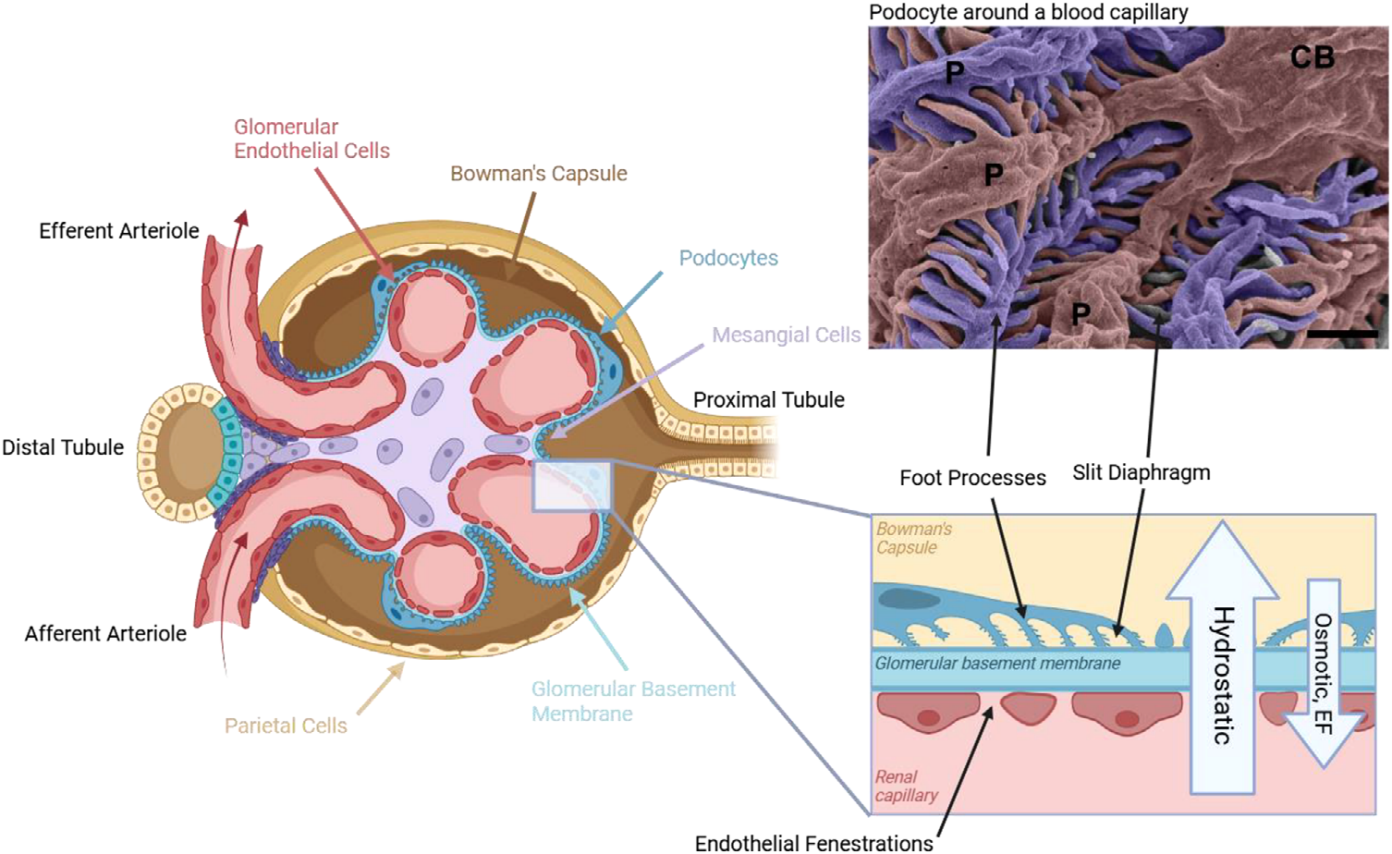
Scheme of the human glomerulus with a focus on the glomerular filtration barrier, presenting the phenomenon responsible for kidney filtration. (Created in BioRender. Perry, G. (2025) https://BioRender.com/y90e964) The scanning electron microscopy image shows two podocytes in the Bowman's Capsule (Reproduced with permission under terms of the CC‐BY license ^[^ ^13^ ^]^. 2015, Nature‐Springer), (scale bar = 1 µm, EF = electrical forces).

Each kidney is composed of approximately one million functional units called nephrons, even if it may vary greatly individual to individual.^[^ ^12^ ^]^ The nephron consists of several key components, including the glomerulus, proximal convoluted tubule, loop of Henle, distal convoluted tubule, and collecting duct. The glomerulus is a capillary network located within the Bowman's capsule. The word “glomerulus” comes from the latin *“glomus”* which means “ball of yarn” as a reference to its intertwined capillary network. Blood enters the glomerulus via the afferent arteriole, and the glomerular filtration barrier selectively filters small molecules like water, glucose, electrolytes, and waste products. Most of it is later returned to the body in the proximal tubule. This filtration barrier is composed of three layers: endothelial cells on the capillary side, podocytes on the urinary side, and they are separated by the glomerular basement membrane (GBM), an extracellular matrix (**Figure** **1**).

In a healthy kidney, the glomerular filtration is the result of several phenomena creating a filtration pressure of about 10–15 mmHg (1333–2000 Pa). The main phenomenon at play is the hydrostatic pressure of the capillary, in competition with the hydrostatic pressure in the Bowman's Capsule (**Table** **1**).^[^ ^14^, ^15^, ^16^ ^]^ The role of electrical forces is still not fully understood.

**Table 1:** The glomerular filtration is the result of several forces facilitating or opposing the filtration.

Phenomenon	Origin	Facilitates/ Opposes filtration
Hydrostatic pressure in the capillary	Pumping of the heart	Facilitates
Hydrostatic pressure in the Bowman's capsule	Glomerular filtration	Opposes
Osmotic pressure	Imbalance in protein concentration as they are retained in the capillaries	Opposes
Electrical forces	The negatively charged glycocalyx retains cations. Consequently, small anions such as Cl‐ pass through slightly faster, creating a potential difference between Bowman's space and the capillary. This generates an electrophoretic flux, which hinders the passage of larger negatively charged molecules.^[^ ^17^ ^]^	Opposes filtration of bigger negatively charged molecules

Due to its complex structure, the barrier freely filters water and molecules smaller than 15 kDa (or with a diameter less than 4 nm). Molecules larger than 15 kDa are increasingly restricted, with those ≈60 kDa being strictly excluded from passing through.^[^ ^18^ ^]^ Moreover, the charge of the molecule has a significant impact, as negatively charged molecules are among those that cannot pass through the barrier. One of the main clues of renal damage is the excessive presence of protein in the urine, called proteinuria, indicating an increase in permeability and therefore an injury to both glomerular cells and/or to the GBM that may lead to kidney disease.^[^ ^19^ ^]^

In order to later understand the design of MPGFB, defining first the main characteristics of the components of the human GFB is a crucial step.

#### 2.1.1 Cells of the GFB

Studies about the GFB and MPGFB have focused on 2 main cell types: podocytes and endothelial cells. Even if those cells are the front line of the filtration, we will see here that they are not the only cells in the glomerulus.

##### Podocytes

Podocytes are unique highly differentiated epithelial cells. They have been extensively studied for some time. They possess a large body that extends to long primary processes and finger‐like projections called foot processes, hence their name. These latter are linked together by a thin porous protein‐based layer called a slit diaphragm that is 30–50 nm wide.^[^ ^20^ ^]^ This structure is crucial for protein retention and defects in the slit diaphragm often lead to proteinuria.^[^ ^21^ ^]^ Their surface is negatively charged due to the presence of anionic proteins^[^ ^22^ ^]^ and some of their main biological markers are podocalyxin, synaptopodin, nephrin, and podocin.^[^ ^23^ ^]^ We are born with a finite number of podocytes which decreases throughout our life, sometimes resulting in renal diseases.^[^ ^24^ ^]^ For further information, the reader can refer to the review.^[^ ^25^ ^]^

##### Endothelial Cells

Glomerular endothelial cells (GEnCs) play a major role in the GFB due to their unique geometry. They possess a structure involving small pores called fenestrations that have a diameter of 60–80 nm.^[^ ^26^ ^]^ Moreover, endothelial cells are coated with a layer of glycoproteins called glycocalyx. It is approximately 200 nm thick and composed of proteins such as proteoglycan and sialoproteins. It is negatively charged, therefore adding again a charge selection to the size one.^[^ ^27^, ^28^ ^]^ The reader can have more information with the reviews.^[^ ^26^, ^29^ ^]^

##### Mesangial Cells

Mesangial cells are smooth muscle‐like cells surrounded by capillaries within the glomerulus. They provide structural support for the glomerular capillary loop. They are also able to contract and alter the blood flow, therefore having a regulatory role over it.^[^ ^30^, ^31^ ^]^ Mesangial cells are specialized pericytes as they are in direct contact with endothelial cells at the paramesangial area. A cross‐talk between mesangial cells and podocytes or endothelial cells has been shown, especially during proteinuria.^[^ ^32^, ^33^ ^]^

##### Parietal Epithelial Cells

Parietal epithelial cells form a tight layer along the inner side of Bowman's capsule. They form a barrier between the filtrate in the Bowman's capsule and the surrounding tissues by preventing leakage. However, their role is not fully understood yet.^[^ ^34^, ^35^ ^]^ Parietal cells, as well as mesangial cells, remain understudied compared to podocytes and endothelial cells.

Those structures, alongside the GBM's particular structure that is discussed below, contribute to the permselectivity of the barrier. They are strongly dependent on each other by being involved in a very complex crosstalk.^[^ ^33^ ^]^

#### 2.1.2 The Glomerular Basement Membrane

Basement membranes (BM) are specialized extracellular matrix (ECM) found beneath epithelial and endothelial cells in various tissues. It provides support for cells and tissues, as well as a pathway for signaling. The GBM is an essential part of the GFB, but its thinness and complex composition make it difficult to reproduce in MPS, as we will highlight in this section.

**Figure 2:**
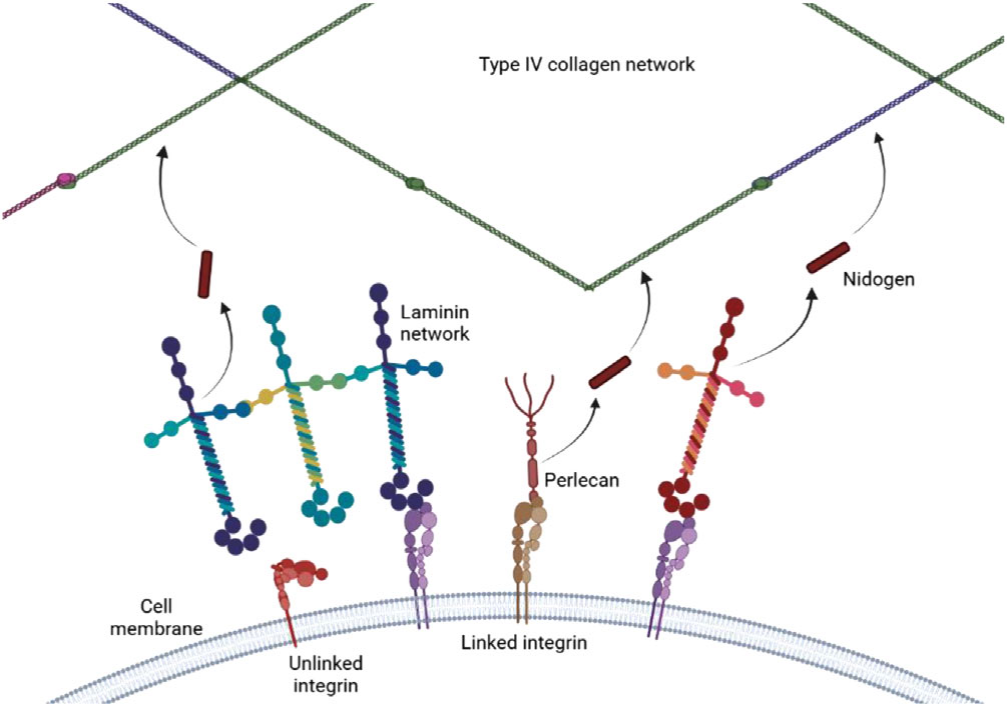
Structure of the in vivo basement membrane is composed of two predominant networks of type IV collagen and laminin. These networks are cross‐linked by nidogen and interact directly, or through proteins such as perlecan, with the cell receptors (here: integrins). The colors indicate different isoforms of the same protein. Created in BioRender. Perry, G. (2025) https://BioRender.com/y90e964, inspired by Perry et al.^[^ ^44^ ^]^ Please note that all schemes are for understanding purposes and the relative sizes of the different components might not be accurate.

The GBM is a thin layer measuring ≈200–300 nm in thickness.^[^ ^36^ ^]^ It primarily consists of type IV collagen, along with other essential proteins like proteoglycans and laminin (**Figure** **2**). Unlike most collagens, type IV collagen does not form fibrils but instead polymerizes into a network as we will see in the next paragraph^[^ ^37^, ^38^ ^]^ which adds an additional layer to the size barrier. Furthermore, the presence of negatively charged proteoglycans, with their glycosaminoglycan chains, was thought to contribute to the charge selectivity of the barrier.^[^ ^27^ ^]^ However recent work has shown that the GBM is not a significant actor of the charge selection.^[^ ^39^ ^]^ The laminin protein plays a crucial role in the GBM integrity by forming a network without which the type IV collagen network cannot assemble in the embryonic stages.^[^ ^40^ ^]^ During glomerulogenesis, endothelial cells and podocytes both produce GBM and the layers fuse together. They continue producing GBM throughout our life.^[^ ^41^ ^]^ The complex composition of the GBM, and more generally of the ECM of glomerular cells, has been extensively studied by Rachel Lennon and her collaborators using mass spectrometry‐based proteomics.^[^ ^42^, ^43^ ^]^ We present here the main components.

##### Type IV Collagen

There are 28 types of collagens in the human body. The most prevalent is type I collagen, representing 90% of all and mainly found in the skin. However, all types are crucial for the integrity of the body. Type IV collagen is a peculiar form of collagen present in basement membranes. The sub‐unit of the collagen is called an α chain, six distinct α chains exist, from α1(IV) to α6(IV), themselves composed of around 1700 amino‐acids arranged in the repetition of the sequence Gly‐X‐Y.^[^ ^45^ ^]^ Each molecule of collagen, or protomer, is composed by the assembly of three α chains, but only three configurations are possible: α1α1α2, the most abundant, α3α4α5, and α5α5α6.^[^ ^46^ ^]^ The protomer is composed of the central triple helix, as well as two non‐collagenic domains on both ends: the N‐ter domain, called 7S, and the C‐ter domain, called NC1. Four 7S domain can assemble, and two NC1 can bound together as well, forming a sheet‐like polymeric network specific of type IV collagen, first studied by electron microscopy by Yurchenco and Ruben.^[^ ^37^ ^]^ Another particularity of type IV collagen is the occasional interruptions in the Gly‐X‐Y sequence (21 to 26 depending on the α chain) that serves, among other, as cells binding sites, as well as intra and inter‐chains bonds.^[^ ^45^, ^47^ ^]^ It is important to note that depending on the development state of the glomerulus, the composition of the type IV collagen regarding α chains will change. In the embryonic stages of renal development, only the chain α1α1α2 is present, then as the glomeruli matures, the chains are replaced by the isoform α3α4α5 secreted by the podocytes. Failure in this replacement can lead to renal defects.^[^ ^39^ ^]^

##### Laminins

Laminin is a family of heterotrimers glycoproteins composed of the chains α, β and γ. They are responsible, amongst other things, for structural support by assembling into a sheet‐like polymeric network, like type IV collagen, via the N‐ter of αβγ chains mediated by Ca^2+^ ions.^[^ ^48^, ^49^, ^50^, ^51^ ^]^ In GBM, the main isoform in the GBM is laminin‐α5β2γ1, however, at embryonic stages, laminin is present in the isoform α1β1γ1, which transitions into α5β1γ1 then α5β2γ1, each form being crucial as a mutation in either one of them results in genetic diseases.^[^ ^52^ ^]^

##### Proteoglycans

Proteoglycans are a subclass of glycoproteins, where the carbohydrate is a glycosaminoglycan (i.e., a polysaccharide constituted of repeating disaccharide units). There are many proteoglycans in the GBM, the main ones being agrin and perlecan. Their principal function is to cross‐link the components of the GBM, especially the networks of collagen IV and laminin, along with the glycoprotein nidogen.^[^ ^53^ ^]^ They also play a role in the elasticity of the membrane.^[^ ^54^ ^]^

##### Integrins

Integrins are proteins that play a crucial role in cell adhesion. There are 24 types of integrins each composed of two subunits α (120–180 kDa) and β (90–110 kDa). They are located in the cell membrane and have the ability to bind to ligands specific to the type of integrin (RGD, GFOGER …) assuring the adherence of cells on the ECM.^[^ ^55^ ^]^ They are also responsible for inside‐out and outside‐in signaling as they possess two states: active, in which the protein have high affinity to the ligand, and inactive, where the protein will not bind the said ligand. GEnCs express α1β1, α2β1, α3β1, α5β1, αvβ1, and αvβ3 integrins.^[^ ^56^ ^]^ Regarding podocytes, the main integrin is α3β1, its absence specifically in podocytes results in the inability to form mature foot processes.^[^ ^57^ ^]^

As said, the GFB is a complex assembly of components. This section intended to give a broad overview to highlight its main characteristics. For further investigations on the components of the GFB, we advise the reader to consult the following reviews.^[^ ^14^, ^58^ ^]^

### 2.2 Physiopathology

CKD is a progressive condition marked by the gradual loss of kidney function, often driven by systemic diseases like diabetes and hypertension. These conditions significantly impact the kidneys, leading to complex pathophysiological changes that contribute to CKD's development. Diabetic nephropathy, one of the leading causes of CKD, affects nearly 40% of people with diabetes and is characterized by increased protein excretion in urine and reduced glomerular filtration rate.^[^ ^59^ ^]^ The relationship between hypertension and kidney disease is complex, with high blood pressure both contributing to and resulting from kidney dysfunction.^[^ ^60^, ^61^ ^]^ This interplay is further complicated by the increased risk of cardiovascular diseases in CKD patients, with more patients dying from cardiovascular complications than progressing to end‐stage renal disease.^[^ ^62^ ^]^ Additionally, as kidneys filter blood, they are first in line to suffer from drug‐induced kidney injury, a significant contributor to CKD. Nephrotoxic drugs such as aminoglycosides, Adriamycin, and nonsteroidal anti‐inflammatory drugs (NSAIDs) are particularly harmful due to the kidneys' role in filtering and excreting these substances. For instance, aminoglycosides can accumulate in the kidney's proximal tubules, causing lysosomal rupture and tubular dysfunction, while Adriamycin can impair the glomerular filtration barrier, leading to proteinuria and further kidney damage.^[^ ^63^ ^]^

When the kidneys cannot properly function anymore, dialysis machines need to take over, with very limited life expectancy – 5.4 years according to the French “Haute Autorité de Sante”.^[^ ^64^ ^]^ Around 2 million people in the world are on dialysis, and this number likely represents less than 10% of those who need it.^[^ ^65^ ^]^ When patients reach the end stage of renal failure, the only viable option to sustain their lives is dialysis while awaiting a kidney transplant, provided they are eligible. According to the Global Observatory on Donation and Transplantation, an estimated 92 500 kidney transplants occurred worldwide in 2021. However, this life‐saving procedure is disproportionately distributed among countries, revealing global inequalities.^[^ ^66^ ^]^ The United States leads the world in kidney transplants, accounting for nearly one‐fourth of all cases. Despite this, over 90 000 individuals are on the kidney transplant waiting list in the US, comprising 80% of all organ transplant candidates. The average waiting time is 3 to 5 years, highlighting the urgency of further research to slow or halt the progression of kidney diseases, reduce the demand for transplants, and enhance the quality of life for patients.^[^ ^67^ ^]^

Overall, the development of CKD is driven by a combination of systemic diseases and external factors like drug toxicity, all of which contribute to the progressive decline in kidney function. All of those diseases have complex pathways, affecting kidneys on their own way. While a more detailed overview of kidney diseases will be given in another section, we can already highlight the need for kidney models to further study those pathologies. 2D cell culture is widely used but poorly replicates human renal physiology, and in vivo models have inherent limitations. Very promising is the emergence of new culture techniques, including 3D cell culture, dynamic conditions, and a more controlled experimental environment. GFB is no exception, and the following sections will present the advances made in MPGFB.

## 3 Development of Glomerulus Models

To achieve a better understanding of these diseases, there is a pressing need for improved research models. In vivo models are widely utilized because in vitro models struggle to replicate the intricate interplay and mechanisms occurring within living organisms. However, in vivo models also have limitations, given the fundamental differences between animals and humans, including variations in metabolism. Moreover, ethical concerns related to animal testing are on the rise, prompting the adoption of the 3Rs principle: Reduce, Replace, Refine. The primary objective is clear: to minimize or, if possible, eliminate animal experimentation, and when necessary, reduce it to the minimum extent while ensuring ethical treatment. In this context, microfluidics, and MPS in general, offer significant promise in providing both better physiological relevance and reducing the need for animal experiments.

**Figure 3:**
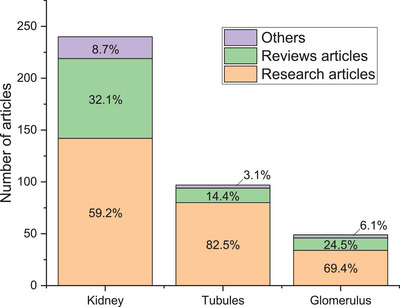
Number of articles in PubMed regarding Kidney‐on‐chip VS Tubule‐on‐chip and glomerulus‐on‐chip. The percentage is the repartition of the type of article for each category (Kidney, tubule, or glomerulus). The methodology is detailed in SI. Distal and proximal tubules were not differentiated. We note the absence of other parts of the kidney such as the collecting duct or the loop of Henle.

MPS can be divided into different categories: spheroids, organoids, and organ‐on‐chip. Spheroids are 3D cellular aggregates formed by culturing cells in suspension, promoting aggregation. They are easily formed and cheap, but lack the complex architecture of tissues and organs. Organoids on the other hand are self‐organized 3D structures that can be derived from stem cells or tissue samples. They replicate some aspects of organ function and structure. They offer valuable models for studying organ development, disease modeling, and drug screening. However, they lack vascularization and it is complicated to analyze the cells inside of the aggregate without destroying the organoid. Lastly, organ‐on‐chips aim to replicate the structural and functional complexity of an organ within a controlled environment by placing cells in microchannels. The term “organ‐on‐chip” encapsulates the ambition to mimic the complexity of whole organs within microfluidic devices. However, it is important to acknowledge that current technology often focuses on replicating specific functions or physiological features of organs rather than fully reproducing their entirety. The kidney is a complex organ with multiple functional units, and whereas some of its units such as the tubule have been explored in term of MPS, the glomerulus is lagging behind (**Figure** **3**).^[^ ^68^ ^]^ While these systems offer valuable insights into organ‐level responses, they may not encompass the full spectrum of organ functionality. Thus, while the name may imply a comprehensive replication, it is more accurate to view organ‐on‐chip systems as sophisticated tools that capture specific aspects of organ physiology within a controlled microenvironment. In this review, we will only focus on organs‐on‐chip, and we will refer to them as MPS when possible.

A lot of work has been turned towards the renal proximal tubule, and less on the glomerulus.^[^ ^68^ ^]^ In this second section, we present the materials and technologies employed to replicate various components of the GFB to build MPGFB.

### 3.1 Cell Types Used in MPGFB

#### 3.1.1 Cell Types

In all cell‐related experiments, one must ask which cell type to use. Until recently, all MPGFB only contained GEnCs and podocytes. As we have seen previously in this review, these are not the only cells present in the glomerulus as there are also mesangial and parietal cells. Recently, Pajoumshariati et al.^[^ ^69^ ^]^ have published the first device to our knowledge combining GEnCs, podocytes, and mesangial cells. In this section, we will focus only on GEnCs and podocytes. There are a lot of possibilities when it comes to choosing which cells to use, some are more relevant than others, better characterized, or easier to use. We will review the various choices researchers have made in MPGFB (**Table** **2**).

**Table 2:** List of the origin of the cells used in MPGFB.

Cell type	Origin	Ref
Glomerular endothelial cells	Animal	[70, 71, 72, 73]
	Human Immortalized	[74, 75, 76]
	Human primary	[69, 77, 78, 79, 80]
	Human iPSCs	[81, 82]
	Other (HUVEC)	[83, 84, 85]
Podocytes	Animal	[70, 71, 72, 73, 75, 86, 87, 88]
	Human Immortalized	[74, 76, 77, 79, 84, 85, 89]
	Human primary	[78, 79, 80]
	Human PSCs	[69, 77, 78, 79, 81, 82, 83]

##### Mammal Cells

Humans and animals share a significant portion of their DNA, especially with mammal, making animal cells a valuable tool for the study of diseases. Furthermore, researchers can precisely control the genetic makeup of laboratory animals and induce genetic diseases to investigate their effects. Using animal cells also offers the advantage of having control over the animal's environment, including factors such as diet, exercise, and disease exposure, which allows for targeted research on specific factors using these cells. However, it is crucial to acknowledge that animal cells have their limitations, as each species possesses unique characteristics and differences.^[^ ^90^ ^]^ The comprehensive review^[^ ^91^ ^]^ highlights those differences. Therefore, even if the animal‐originated cells used in MPGFB are mammal cells (usually murine), only human cells can truly reflect the physiological aspects of the human body and animal cells are not satisfying models when it comes to human disease modeling.^[^ ^92^ ^]^

##### Immortalized Cell Lines Versus Primary Cells

Immortalized cell lines refer to cells that have been modified to halt their degenerative process, or senescence, a term derived from the Latin *“senex”*, meaning “old.” These mutations can occur spontaneously, as seen in some tumor cells, or they can be induced artificially. Immortalized cell lines offer several advantages, including well‐documented characteristics, relatively stable behavior over time, and rapid proliferation. As a result of their mutation, they lack of physiological relevancy. On the other hand, primary cells are directly sourced from the tissue of interest, offering the highest level of physiological relevance. However, primary cells tend to be less homogeneous, which can lead to greater variability in experimental results. They must be used relatively quickly since their senescence process remains active, and their characteristics may change over successive passages, ultimately leading to cell death. Additionally, as mentioned above, primary cells come from the tissue of interest, but are sourced from ill patients. Even if the cells do not come from affected organs, we cannot be sure that they have not been indirectly affected by the disease or disease markers.

A lineage of immortalized podocytes AB8/13 was developed only about twenty years ago by Peter Mundel's team.^[^ ^93^ ^]^ This lineage is still the only one to our knowledge. Regarding glomerular endothelial cells, an immortalized cell line expressing fenestrations (Conditionally Immortalized Glomerular Endothelial Cells) was developed around the same time.^[^ ^94^ ^]^ This delay in the development of immortalized cell lines has partly contributed to the lesser study of the glomerulus compared to other parts of the kidney, such as the tubule.

Both immortalized cell lines and primary cells are used in MPGFB research, particularly for endothelial cells and podocytes. The choice between these cell types depends on the specific research goals and trade‐offs between physiological relevance and experimental convenience.

##### Human Induced Pluripotent Stem Cells (hiPSCs)

Sinya Yamanaka and John Gurdon were awarded the Nobel Prize in Medicine in 2012 for their groundbreaking discovery of a method to reprogram mature cells into pluripotent stem cells, thereby eliminating the need for embryonic stem cells. In contemporary research, iPSCs have become an indispensable tool due to their ability to provide the most relevant experimental system for studying cellular differentiation at all stages. Nevertheless, it is important to note that iPSCs are challenging to handle. The differentiation protocols required are often complex and time‐consuming, and these cells are highly sensitive to their microenvironment. Moreover, depending on the reprogramation protocol, iPSCs may respond differently to the same differentiation process. While differentiation protocols for endothelial cells have been known for a long time and are available commercially, the situation is different for GEnCS and podocytes. Although several differentiation protocols exist to generate podocytes from iPSCs^[^ ^95^, ^96^, ^97^, ^98^ ^]^ in culture well plate, they all display a major drawback by using batch‐to‐batch dependent and animal‐based products (Fetal Bovine Serum or growth factor‐reduced Matrigel). Musah et al.^[^ ^99^ ^]^ established a protocol where the final differentiation step is performed within a microfluidic device and in coculture with endothelial cells providing a more physiologically relevant microenvironment for the maturation in podocytes. For these reasons, this protocol is now widely used in MPGFB.^[^ ^69^, ^77^, ^81^, ^82^ ^]^

##### Cells Sourced From Organoids and Spheroids

A new source of cells has emerged recently using MPS technology. It is possible to grow spheroids/organoids and retrieve the cells to place them into organs‐on‐chip. To our knowledge, two MPGFB use this technique.^[^ ^70^, ^83^ ^]^ However, the dissociation process is quite complicated and not without consequences on the cells. After the dissociation of the organoids, the cells must be sorted in order to only keep the cells of interest.^[^ ^100^ ^]^ This pathway often results in little amount cells, even if it allows a great control over the cells environment. For example, Sharmin et al.^[^ ^101^ ^]^ inserted a GFP cassette upstream of the nephrin (NPHS1) start codon to sort podocytes sourced from organoids.^[^ ^102^ ^]^ The sorting yield is low around 7.45% of the initial cell population, moreover, only 94% of the sorted podocytes are double positive for nephrin and podocin using antibodies.

##### Choosing the Right Cells

Whereas podocytes are quite unique epithelial cells, endothelial cells are present everywhere in the body. Therefore, in the literature instead of GEnCs, Human Umbilical Vein Endothelial Cells (HUVECs) are sometimes used, putting aside the particularities of GEnCs seen in the 1st section.

Additionally, regardless of the cell type, the often‐overlooked factor of sex‐specific characteristics of these cells should be considered.^[^ ^103^ ^]^ Despite the apparent lack of differences between male and female kidneys, growing evidence suggests its significance^[^ ^104^, ^105^, ^106^ ^]^ and yet very few articles even mention it.^[^ ^78^ ^]^ It is especially true in some diseases, such as Alport Syndrome (AS), where the chromosome X suffers from a mutation, therefore affecting more men than women.

#### 3.1.2 Markers of Glomerular Cells

To assess cell behavior, it is essential to monitor specific marker levels. When comparing two systems, optimal performance is typically linked to cells expressing the highest levels of these markers, which are often quantified through immunofluorescence (IF) or PCR, ensuring they are located in the expected regions. The main markers of glomerular cells are listed in the following table. Markers can be either proteins or the associated genes. While podocytes are kidney‐specific cells, endothelial cells are not limited to the kidney. It is important to recognize that most of the markers used for identifying GEnCs are specific only to endothelial cells in general, not those specifically in the glomerulus. Although defining specific makers to GEnCs is no easy task, the following markers are reported to be specific to GEnCs compared to other glomerular cells: PLAT, EMCN, TSAPN7, EHD3, LPL, HECW2, KDR, and SOST.^[^ ^107^, ^108^ ^]^ They have been identified either by single cell RNA‐sequencing, or single nuclei RNA‐sequencing, or both within renal cell population. To our knowledge, amongst those markers only EHD3, a fenestration regulator,^[^ ^109^ ^]^ is studied in MPGFB.^[^ ^69^, ^78^, ^79^ ^]^ It would be advantageous to employ markers tailored to GEnCs to accurately assess their distinct behavior.

The main cell markers studied in MPGFB are listed in **Table** **3**. They can be assessed either by IF, PCR, flow cytometry, western blot, or other means of analysis.

**Table 3:** Main markers used for glomerular cells in MPGFB.

Cell type	Protein (GENE)	Type	Localization
Podocytes	Podocin (NPHS2)	Transmembrane protein	Foot processes/Slit diaphragm
	Nephrin (NPHS1)	Transmembrane protein	Slit diaphragm membrane
	Podocalyxin (PODXL)	Transmembrane protein	Glycocalyx
	Synaptopodin (SYNPO)	Actin‐binding protein	Cytoplasm
	WT1 Transcription Factor (WT1)	Gene expression regulation protein	Nuclei
Endothelial cells	Platelet Endothelial Cell Adhesion Molecule 1 (PECAM1, CD31)	Transmembrane protein	Cell membrane
	Vascular Cell Adhesion Molecule 1 (VCAM1, CD106)	Transmembrane protein	Cell membrane
	Vascular Endothelial Cadherin (VE‐cadherin, CD144, CHD5)	Cell adhesion transmembrane protein	Cell junction
	von Willebrand Factor (vWF)	Endothelial damage	No specific localization
Glomerular endothelial cells	EH Domain Containing 3 (EHD3)	Protein	Cell membrane

### 3.2 Glomerular Basement Membrane

The GBM is a highly complex membrane, as illustrated in a previous section. Consequently, many researchers opt to replace it with alternative components that vary in physiological relevance. One approach to circumvent this challenge is to immobilize GFB cells and allow them to produce the GBM. Researchers also face the decision of whether to preserve the glomerular special spherical shape in 3D models, and possibly introduce vascularization, which can complicate the analysis of cells and markers. Alternatively, they may choose to make concessions in favor of gaining more insights into cell behavior by using 2D models.

#### 3.2.1 Cell‐Produced GBM

**Figure 4:**
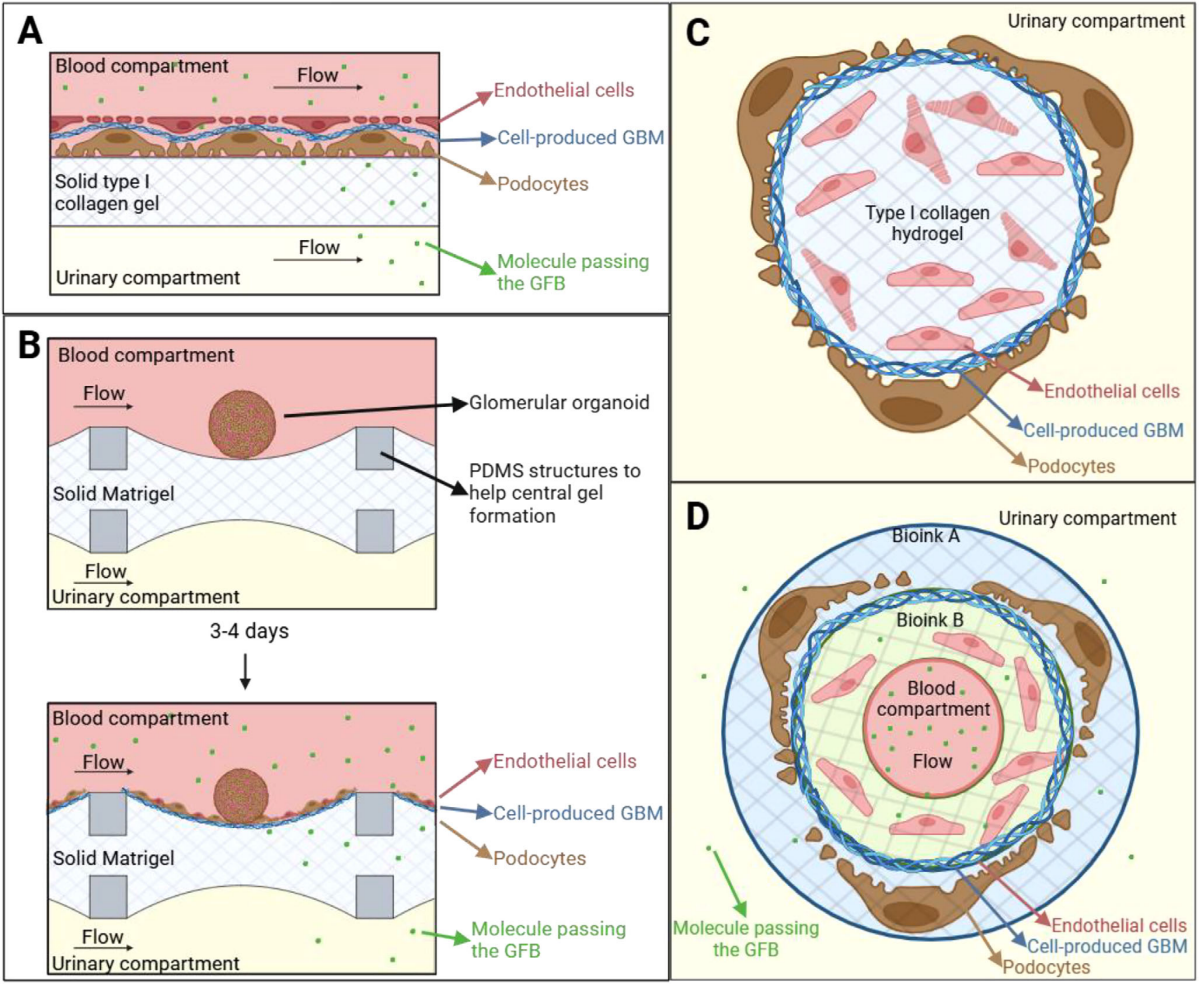
Devices with a GBM produced by the cells. The grid represents the solid parts of the device. Please note that all scheme are for understanding purposes and the relative sizes of the different components might not be representative of the real devices. A: podocytes are first deposited on a solid hydrogel, and then endothelial cells are seeded on top of the podocytes (inspired from).^[^ ^79^, ^110^ ^]^ B: glomeruli are seeded on a solid hydrogel and let for a few days in order to form a GFB (inspired from).^[^ ^70^ ^]^ C: a fiber containing endothelial cells embedded in type I collagen is seeded with podocytes on top, there is no flow in this device (inspired from).^[^ ^74^ ^]^ D: a perfusable fiber is made with two bioinks (decellularized porcine kidney ECM) containing either endothelial cells or podocytes. The flow is directed toward the reader (inspired from).^[^ ^80^ ^]^ Created in BioRender. Perry, G. (2025) https://BioRender.com/y90e964.

A first approach to establishing a GBM involves inducing cells to produce it, mirroring in vivo conditions. This has been achieved by initially seeding podocytes on a flat surface and subsequently seeding endothelial cells on top of the podocytes (**Figure** **4**A).^[^ ^79^, ^110^ ^]^ An alternative method was used by Wang et al.,^[^ ^70^ ^]^ who seeded entire rat glomeruli organoid on Matrigel, allowing them to spread and form a GFB on the Matrigel (Figure 4B). It is worth noting that even if the presence of podocytes, endothelial cells, and ECM proteins was confirmed in their GFB, their respective organization into 3 layers cannot be ensured. Another alternative is embedding cells in a hydrogel such as type I collagen permitting to mold them into any shape. It has been shown that GBM components are produced at the frontier between an GEnCs‐containing hydrogel and podocytes (Figure 4C) or podocytes‐containing hydrogel (Figure 4D).^[^ ^74^, ^80^ ^]^ However, these techniques have inherent limitations, as the materials used for cell seeding/embedding lack physiological relevance, failing to reproduce the in vivo podocyte environment accurately. Moreover, these materials can interact with and retain certain molecules, potentially affecting permeability measurements. In any case, to verify the production of the GBM it is essential to evaluate production levels of GBM proteins such as type IV collagen and laminin 521.

#### 3.2.2 Membrane Replacement

**Figure 5:**
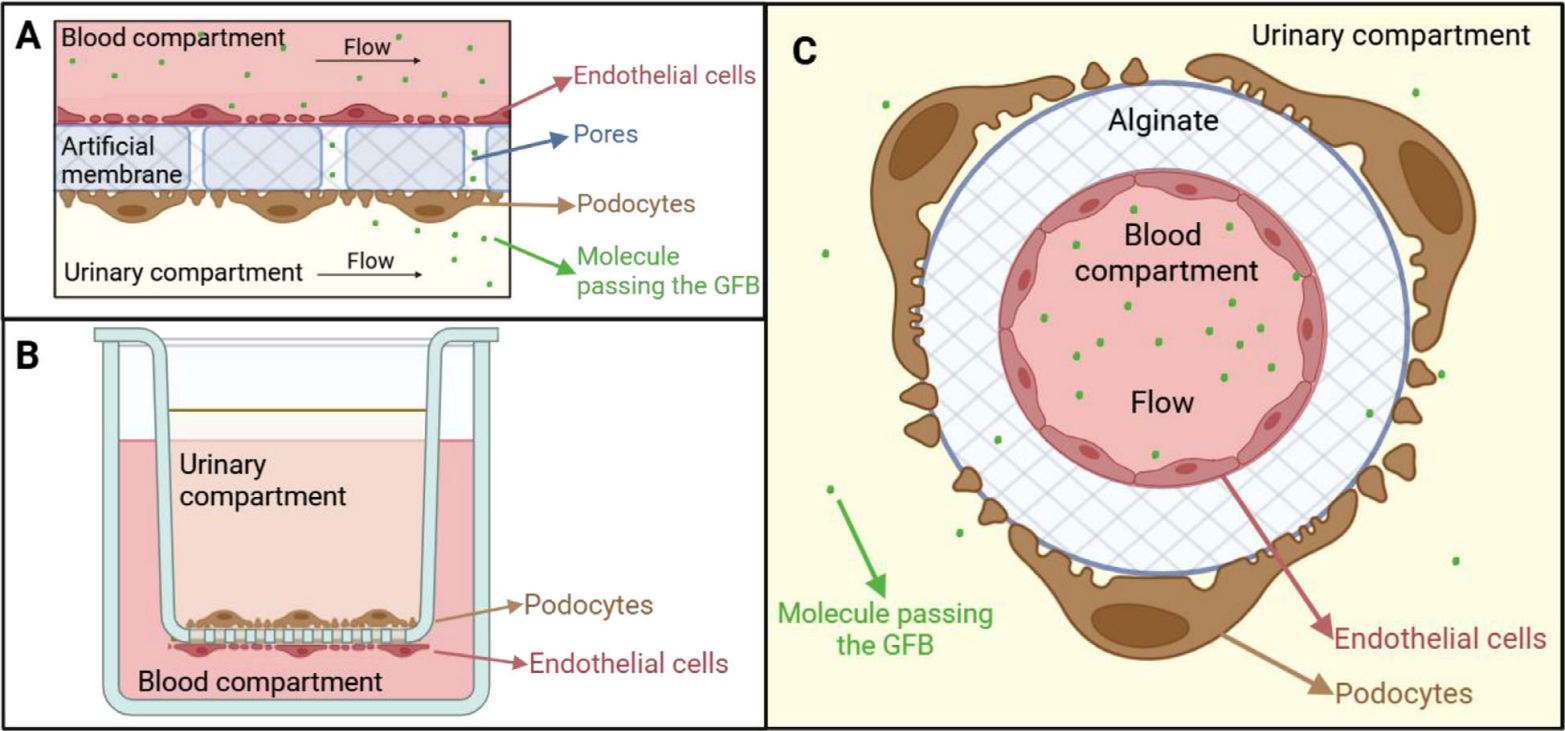
Devices with a GBM replaced by an artificial membrane. The grid represents the solid parts of the device. Please note that all scheme are for understanding purposes and the relative sizes of the different components might not be representative of the real devices. A: main geometry of this type of devices, where endothelial cells are separated from podocytes by a porous membrane. This membrane may also be naturally permeable, in which case there is no need for pores. B: Transwell with endothelial cells and podocytes on each side of the permeable membrane. C: perfused alginate fiber with endothelial cells seeded inside and podocyte outside. The flow is directed toward the reader (inspired from^[^ ^73^ ^]^). Created in BioRender. Perry, G. (2025) https://BioRender.com/y90e964.

One may choose to replace the GBM with an alternative material and seed cells on both sides, which is the main geometry of MPGFB (**Figure** **5**A). This synthetic membrane should possess porosity, whether it is artificial, by adding pores into the material, or natural by using permeable materials. The selected material also has to be non‐cytotoxic and ideally closely resemble the in vivo composition to ensure the physiological relevance of cell behavior. Furthermore, it should be sufficiently rigid to support cell adhesion and development. However, reproducing the thinness of the in vivo GBM, which is approximately 250 nm,^[^ ^36^ ^]^ has proven challenging. Synthetic GBMs are typically much thicker by several orders of magnitude (see Table 5), and some incorporate artificial pores that do not align with the physiological anatomy of the GFB, potentially affecting permselectivity measurements and alter intercellular crosstalk. Transwells can also be used as they already possess a porous membrane (Figure 5B). However, making transwells perfusable can prove challenging, even if it has been done.^[^ ^89^ ^]^ To tackle the thickness issue, Kim et al.^[^ ^88^ ^]^ built an MPGFB where the thickness of a Matrigel‐based GBM can be tuned. They managed to achieve thicknesses from 2 to 5 µm even if they found no significant influence on the permeability. However, the chip is complicated to develop as they report the GFB fails to form almost half the time. Mou et al.^[^ ^82^ ^]^ have reported the fabrication of a thin (3.5 µm) membrane made by electrospun silk fibroin and induced conformation change from random coil to β‐sheets by immersing them in methanol. Xie et al.^[^ ^73^ ^]^ had an interesting approach where they created an alginate perfusable fiber and seeded endothelial cells inside and podocytes outside (Figure 5C). However, their fiber remained thick (30 µm) compared to other devices.

The characteristics of some materials are discussed below (**Table** **4**) in regards to the application, here the reproduction of a porous membrane mimicking the GBM.

**Table 4:** Pros and cons of the commonly used materials for ECM replacement.

Material	Advantages	Inconvenients
Polydimethylsiloxane (PDMS)	Easy fabrication and integration	Absorbs small hydrophobic molecules Not physiologic Not porous
Polycarbonate track etched (PCTE)	Commercial Easy integration	Not physiologic Not porous
Matrigel	Commercial material Composition close to BM's	Not specific to one BM Composition varies batch to batch Produced by mouse sarcoma cells
Collagen I	Present in the body Easy integration	Not the main component of BM's Not the same porosity as BM's
Collagen IV‐based hydrogel	Closer composition to BM's Composition tunable depending on the needs	Difficult to not denature the proteins Difficult to polymerize therefore to integrate Complex to integrate into devices

Additionally, this synthetic membrane is often coated with extracellular matrix components, such as laminin or fibronectin, to enhance cell adhesion. Nevertheless, it is essential to assess the membrane's permselectivity and permeability independently, as it can retain molecules. Ideally, the synthetic membrane should eventually be replaced by cell‐produced ECM, possibly through enzymatic degradation.^[^ ^111^ ^]^ However, as of now, this transition to in vivo‐like conditions has not been observed in MPGFB.

A list of the synthetic membranes used in MPGFB is presented in **Table** **5**. If several membranes or coatings are used in an article, only the one offering the best results according to the article is listed.

**Table 5:** Membrane replacement in MPGFB (N.D. = Not Disclosed).

Refs.	Material	Thickness	Coating	Artificial pores
[75]	Polyester carbonate	10µm	BME Cultrex^TM^	10 µm ϕ
[77, 81]	PDMS	50µm	Laminin 511	7 µm ϕ; 40 µm apart
[71]	N.D.	10µm	Geltrex™ LDEV‐Free Reduced Growth Factor Basement Membrane Matrix	1 µm ϕ
[73]	RGD‐conjugated alginate	30 µm approx.	No	No
[83]	Gel with fibrinogen, collagen IV, laminin 521, aprotinin, and thrombin	500µm	Endo: fibronectin, EBM2 Podo: collagen IV, laminin 521, EGM‐2	No
[84]	Polycarbonate Track‐Etched (Whatman Cyclopore)	20µm	Fibronectin	Yes, 1 µm ϕ
[72]	Polycarbonate Track‐Etched (Whatman Nuclepore )	∼10µm	Polydopamine and collagen I	Yes, 8 µm ϕ
[85]	Polyethersulferone (Sterlitech)	N.D.	Collagen I or laminin or heparin sulfate or collagen IV	30 nm ϕ
[88]	Growth factor reduced Matrigel	2‐5µm	Collagen I, matrigel or poly‐D‐lysine	No
[76]	Polyethylene	30µm	Human fibronectin	2 µm ϕ
[69]	PDMS	50µm	Endo: fibronectin, collagen IV, collagen I (gel containing mesangial cells) Podo: laminin 521	7 µm ϕ; 40 µm apart
[82]	Electrospun silk fibroin	3.5µm	Laminin 511	No, natural high porosity (55.9% void/pore area)

#### 3.2.3 Toward the Nephron on Chip

The GFB is only a part of the nephron, itself one of the several million nephrons in each kidney: we are still far from a real “kidney‐on‐a‐chip”. To our knowledge, only two devices have integrated the filtration of the GFB, and the reintegration of the tubule.^[^ ^71^, ^85^ ^]^ Each has inherent limitations, such as using animal cells or having a syringe‐like shape for the glomerulus. Moreover, these two elements only cannot pretend to recreate the complex functions of a nephron. The vasculature of the nephron is complex, and for now organs‐on‐chip are far from recreating it.

### 3.3 Cultivating Cells in a MPGFB

#### 3.3.1 Factors Impacting Cells

A key advantage of MPS is the control over the cell environment. We will see in this section the numerous factors that could affect cell development, and therefore MPGFB reliability.

##### Single VS Co‐Culture

Most articles reporting MPGFB use fully differentiated cells. However, in vivo, the growth of podocytes and endothelial cells are deeply intertwined, as they both secrete growth factors directed to the other such as Vascular Endothelial Growth Factor (VEGF). ‘t Hart et al.^[^ ^72^ ^]^ showed that the culture of mouse immortalized GEnCs and podocytes in a MPGFB modified their characteristics such as morphology and transcriptome, and particularly thickens the glycocalyx which is a key element in glomerular filtration. Taking mature cells and putting them in a new environment will change their behavior. However, it will never fully reproduce the behavior of cells developing and maturing directly into this environment. Therefore, to obtain the full benefits of co‐culture, iPSCs should be used. As of today, their integration in MPGFB remains a challenge. Roye et al. developed a dispositive where endothelial cells and podocytes were obtained using iPSCs from a single patient.^[^ ^81^ ^]^ However, such a system is difficult to develop as iPSCs differentiation can take a long time (weeks to months depending on the protocol). More tests are needed in order to prove that the iPSCs‐originated podocytes and endothelial cells have significantly more physiologically relevant characteristics than primary ones. It is nonetheless a promising first step towards personalized medicine. Moreover, as it was mentioned before, Pajoumshariati et al. reported the first tri‐culture chip with only glomerulus cells to our knowledge.^[^ ^69^ ^]^ They showed that the addition of mesangial cells modified the transcriptome of podocytes and GEnCs, and improved albumin permeability. They also studied in a very comprehensive way the gene expression of different types of cultures, showing that monocultures express signatures of diseases such as membranous glomerulopathy, once again illustrating the importance of cell cross‐talk.

##### Flow and Pressure

**Figure 6:**
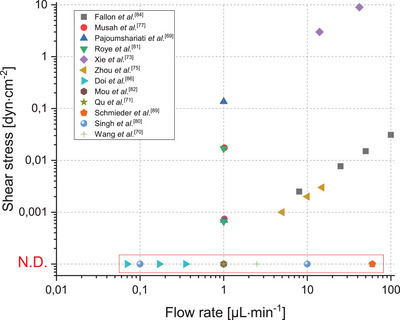
Flow rates and shear stresses used in MPGFB. The detailed data can be found in Table S1 (Supporting Information). In some articles only the flow rate is mentioned, those are indicated in the red box (N.D. = Not Disclosed).

In their natural in vivo environment, cells are exposed to fluid flows, and consequently, experience shear stress (SS). GEnCs endure SS from blood flow. The mean SS ranges from 30 to 50 dyn·cm^−2^ (1 Pa = 10 dyn·cm^−2^) with a large variance throughout the capillary network.^[^ ^112^ ^]^ For podocytes, the SS value is still in discussion as it varies greatly within the glomerulus. In their review, Wang et al.^[^ ^113^ ^]^ mention SS up to 80 dyn·cm^−2^, while other studies shown that SS above 0.25 dyn·cm^−2^ damages podocytes.^[^ ^114^ ^]^ However, it is worth noting that high SS will damage more young podocytes rather than mature ones.^[^ ^69^ ^]^ This uncertainty regarding shear stress can be seen in MPGFB as the shear stresses used vary from several orders of magnitude (**Figure** **6**). It is now fully admitted that cells developing under dynamic conditions show better behavior than in static conditions. This is particularly relevant in the glomerulus, where three distinct flows exist: the blood flow, the primary urine flow, and, in between, the filtration flow. It is crucial for in vitro devices to accurately replicate these shear rates, as they exert a significant influence on cell behavior. This is one of the principal advantages of microfluidic devices, which provide precise control over shear rates. Still, some devices surprisingly do not include flow even if they use microfluidic chips.^[^ ^72^, ^83^, ^88^ ^]^ Furthermore, the direction of the flow also exerts a profound impact on cellular responses. Most in vitro devices use two applied flows representing the blood flow and primary urine flow. These flows are, most of the time, oriented parallel to the cell layer. However, some chose to orient it perpendicularly to the cell membrane, such as Zhang and Mahler^[^ ^85^ ^]^ where they used a syringe‐like shape, which is less physiologically relevant as the molecules are forced through the membrane, and the ones that cannot pass will accumulate at the membrane.

High flow rates are used to model hypertension^[^ ^75^ ^]^ and it has been shown that high flow rates induce cell stress and ultimately damage the barrier. However, one must be careful as the relationship between flow rate and pressure might be sometimes misunderstood. The flow rate is proportionally linked to the pressure differential with the equation:

(1) ΔP=R×Q

With Δ*P* the pressure differential, *Q* the flow rate, and *R* the hydrodynamic resistance that depends on the geometry of the channel and on the fluid properties.

In most cases, the outlet pressure is the atmospheric pressure, and the flow rate in the chip is directly proportional to the pressure at the inlet. However, it is important to keep in mind that low flow rates are achievable at high pressures if the pressure differential is low. In most cases, hypertension is independent of the flow rate, as hypertension mostly relies on the vasoconstriction status of the blood vessel.^[^ ^115^, ^116^ ^]^ Therefore, one must be careful about the relevance of the model used and the means to achieve it. Controlling the pressure differential applied in the device might be closer to hypertension,^[^ ^76^, ^86^, ^89^ ^]^ but it rather creates a model of high shear stress.

In any case, it is crucial to know and control the flow rate, pressure, and shear rate. Modeling is a convenient tool to estimate those values with softwares such as COMSOL.^[^ ^85^ ^]^

##### Continuous Versus Pumpless Flow

There are several options possible to induce flow. A first category necessitates the use of specific equipment, such as pressure pumps, syringe pumps, or peristaltic pumps. This approach guarantees that cells are consistently exposed to media, mimicking conditions within the body. The media can either be recirculating, which makes the nutrient concentration decrease overtime, or fresh media can be used. However, this option can incur significant costs due to the substantial quantities of media required, especially for prolonged experiments. The majority of MPGFB systems employ a continuous recirculating flow using a peristaltic pump.^[^ ^71^, ^76^, ^77^, ^80^, ^82^, ^84^ ^]^

Alternatively, some opt for a pumpless flow, using gravity as a driving force, where the nutrient concentration also decreases overtime.^[^ ^73^, ^78^, ^79^ ^]^ There are two options for implementing this technique: unidirectional or bidirectional flow. While bidirectional flow, usually involving the rocking of the chip back and forth to move the liquid over cells, is more common, it deviates significantly from physiological flow as the shear stress varies greatly over time. It is therefore preferable to have a unidirectional flow. A device allowing unidirectional flow driven by gravity thanks to clever geometry has been reported.^[^ ^117^ ^]^

##### Cell Density

The density at which cells are seeded is a critical parameter. Excessive cell density may hinder their full development and could even lead to cell death. Conversely, too few cells may result in empty spaces, impeding the formation of a permeable barrier. This is particularly significant when dealing with podocytes, as an incorrect cell density can prevent the proper development of foot processes and interdigitations.

**Figure 7:**
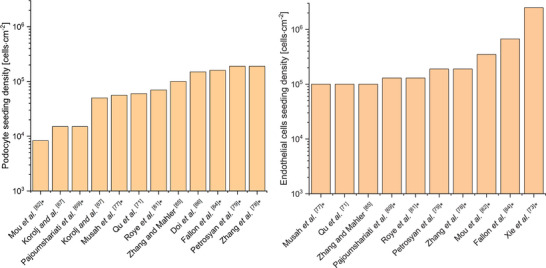
Seeding densities used in MPGFB. *As Estimated with the data of the article, the surface used was the whole surface of the channels, rather than one side only.

It is worth noting that despite being crucial and more useful than the cell concentration used or the number of cells seeded, cell density is not always indicated. When missing, estimating seeding densities from articles can be hard, as all the necessary information to calculate it might not be written. In **Figure** **7**, we tried to calculate the seeding density by estimations of chip surface (*). For all cells, the whole surface is considered (as opposed to the membrane surface only) as they tend to invade the whole channel. Most articles use a density between 10^4^ and 10^6^ cells·cm^−2^. The detailed data can be found in Table S2 (Supporting Information).

##### Support Rigidity

The stiffness of the cell support is known to play a major role in cell attachment and development. The Young's modulus of the external capillary walls of a glomeruli is around 2500 Pa, and is reduced in some diseases.^[^ ^118^ ^]^ Abdallah et al.^[^ ^119^ ^]^ studied podocytes on polyacrylamide surfaces of different stiffnesses and determined that the optimal was between 0.9 and 9.9 kPa. For comparison, PDMS Young's modulus is around 1–2 MPa for a mixing ratio of 10:1,^[^ ^120^ ^]^ highlighting the need for engineering softer surfaces inside of the MPS for cell seeding.

##### 3D Structures

The glomerulus is not a flat, planar surface; in vivo, podocytes are naturally arranged around capillaries and possess microcurvature. Korolj et al.^[^ ^87^ ^]^ demonstrated that when podocytes are seeded on a surface with microcurvature, they express more specific markers, their actin skeleton closely resembles the in vivo structure, and they exhibit a more digitated appearance compared to cells on a flat surface. This underscores the significance of replicating a 3D environment as opposed to a 2D one. An innovative approach was explored by Flegeau et al.,^[^ ^74^ ^]^ where they embedded endothelial cells in a capillary‐shaped hydrogel to mimic blood capillaries and seeded podocytes on top, resulting in a 3D structure. However, this system lacked a flow component. Recently, Singh et al.^[^ ^80^ ^]^ reported building a device with a similar cell‐embedded hydrogel with a capillary‐shape and including flow therefore allowing permeability measurements. Dai et al.^[^ ^76^ ^]^ used twisted hollow polyethylene fibers to make a perfusable capillary‐like scaffold in which cells were seeded. Those two devices reproduce the overall shape of the capillary, but lack the microcurvature. Xie et al.^[^ ^73^ ^]^ also used a perfusable capillary shape but added a glomerulus‐like shape as well as microcurvature thanks to a chemical reaction producing small CO_2_ bubbles. By seeding cells on this scaffold, they showed once again that 3D structures and microtopography improve podocyte development compared to 2D culture.

More specificities can be added to the MPGFB. Musah et al.^[^ ^77^ ^]^ and Roye et al.^[^ ^81^ ^]^ choose to incorporate hollow chambers on the sides of the channel to produce a stretching and relaxation of the membrane, recreating the cyclic pulsations of the blood flow of 1 and 0.4 Hz respectively, enhancing cells’ functional markers such as nephrin.

#### 3.3.2 Evaluating the MPGFB Relevance

Once the chip is made, it is crucial to evaluate its relevance, which means its ability to being a close enough replicate of the in vivo glomerulus. For now, there are no established standards available for evaluating chip performance. Nevertheless, various properties are frequently examined, even in the absence of standardized reference values. As seen previously, cells markers are systematically assessed, but they are not the only evaluated value.

##### Morphology

In addition to cell markers, observing cell morphology provides valuable insights into their proper functioning. For instance, the presence of podocyte foot processes and the fenestrations of GEnCs are essential features. Therefore, it is crucial to verify that these characteristics are indeed exhibited by the cells in the MPGFB. Imaging cells can be challenging depending on the engineering of the MPS. However, a key advantage of organs‐on‐chip over organoids and spheroids is the easier access to all cells since they are not clustered together.

To observe morphology, optical microscopy is straightforward in a simple glass‐PDMS microchip, as the glass used can be a microscope glass slide, enabling easy and quick daily microscopic checks. However, optical microscopy provides limited information. Electron microscopy (EM) can offer more detailed insights into cellular structures, such as endothelial fenestrations or podocyte foot processes. Despite its advantages, using EM in microfluidic devices remains challenging. Only few MPGFB has used EM to demonstrate the induction of relevant morphology.^[^ ^73^, ^74^, ^76^, ^88^ ^]^ The most advanced analysis in a microfluidic device has been made by Mou et al.^[^ ^82^ ^]^ where they demonstrated the apparition of fenestrated GEnCs from iPSCs in the presence of podocytes, as well as the formation of podocyte tertiary foot processes around their silk fibroin‐based membrane.

IF is a routine technique in cell culture and is also commonly used in MPS devices. Thanks to their inherent channels, it is easy to introduce the necessary chemicals for the various steps involved in IF.

##### Inulin Versus Albumin Retention

A classical test used in all MPGFB involves characterizing the retention of molecules with varying sizes. Among these, inulin and albumin are the most frequently utilized. While albumin (66.5 kDa) is recognized for its inability to traverse the GFB due to its larger size, inulin, being considerably smaller (≈4 kDa), can pass freely. Hence, these two molecules represent opposite ends of the permeability spectrum. Another molecule sometimes used is urea (60 Da), being very small it can freely pass. In vivo, the inability of the kidney to filter urea may lead to uremia and eventually death. However, this permselectivity test alone does not provide extensive insight, as it fails to pinpoint the precise threshold of permselectivity. Markers of different sizes and charges should be used to have a fuller comprehension of the membrane. Moreover, there is no gold standard regarding the concentration of these compounds when testing MPGFB, making the comparison between devices difficult.^[^ ^69^ ^]^ Fallon et al.^[^ ^84^ ^]^ developed a platform for real‐time fluorescent monitoring that allows the tracking of fluorescent probes concentration during permeability measurements.

##### The Importance of Controls and Comparison to In Vivo Data

On a broader note, glomerular cells are unique, each possessing specific characteristics. It is important to assess the impact of replacing podocytes and glomerular endothelial cells with non‐glomerular cell types, such as fibroblasts^[^ ^77^, ^79^ ^]^ or human lung endothelial cells,^[^ ^79^ ^]^ particularly in the context of permeability measurements to emphasize the particularity of glomerular cells and ensure their physiological behavior. Only comparing with cell‐free device is not enough to draw conclusions.

While some researchers have attempted to compare their results to in vivo levels,^[^ ^77^, ^87^, ^88^ ^]^ the estimation protocol may yield unsatisfactory results due to the complexity of obtaining in vivo data, which varies significantly between individuals. Moreover, the product of the glomerular filtration is not the urine yet, but the primary urine which has a different composition. The composition of the primary urine is difficult to assess in vivo due to its localization. Therefore, it is necessary to take a step back when comparing the concentrations obtained in MPGFB with in vivo results. To have relevant in vivo comparison, one may explore the proteome and transcriptome of cells while keeping in mind that having a positives RNA expression does not ensure its translation into proteins. However, MPGFB uses small quantities of cells and offers little material for analysis, often not enough for proteome and transcriptome analysis. Despite these challenges, Pajoumshariati et al.^[^ ^69^ ^]^ offered a nice analysis of the transcriptome of their cells, comparing 2D to 3D culture, as well as single, co and tri‐culture, highlighting the limits of monoculture.

Regardless of the cell type used, it is essential to be mindful of its limitations. Achieving a higher degree of relevance often demands more complex and rigorous experimental protocols. Comparing results obtained with different cell lines, considering factors such as cellular morphology, marker expression, permeability, and permselectivity levels is a valuable way to ensure that each cell type's advantages are realized as expected.

##### Sensor‐Based Characterization Methods

The cell phenotype within MPS is mainly monitored by optical microscopy (bright‐field, phase‐contrast, or using fluorescent antibodies). Techniques like PCR, Western‐Blot, and cytometry while providing information about the cell phenotype, are particularly limited when it comes to assessing the complex functional characteristics of MPS due to the low cell amount per system. Therefore, more advanced monitoring methods are necessary to obtain comprehensive insights. Integrated sensors have emerged as simple, real‐time, non‐destructive characterization methods to monitor several parameters such as O_2_ concentration, pH, transepithelial electrical resistance (TEER), metabolites analysis, and more.^[^ ^121^ ^]^ Those sensors can be divided into several categories: mechanical, electrochemical, optical, and electrical.

When working with cells, the biocompatibility of the materials and methods used is a critical requirement. Metals like gold and platinum are often preferred for electrical and electrochemical‐based sensors due to their stability and passive behavior in biological systems. It is vital to use non‐toxic materials ensuring the health and function of cells over extended periods. Moreover, sensor technologies used in MPS must be non‐destructive to maintain the integrity of cells and tissues during monitoring. Real‐time monitoring is particularly crucial in living and evolving systems, as it allows continuous assessment of cellular functions without compromising the biological samples. Given that analyses can extend over several weeks, sensor stability is essential, which poses challenges for the use of enzyme and other bio‐based sensors due to their limited lifespan.

MPGFB belongs to the broader category of MP‐barriers, also including blood brain barrier, or gut for example. To our knowledge, no sensors have been specifically employed in the field of MPGFB. However, sensors have been widely used in the broader domain of MP‐barriers. The most common of these is the TEER measurement, which assesses the cell layer resistance at a single frequency. This technique often requires printed microelectrodes within the device, as the traditional chopstick‐like configuration can produce inhomogeneous current, leading to inaccurate measurements. Impedance measurements, which utilize a broad range of frequencies, can provide more detailed information. Techniques such as Electric Cell‐Substrate Impedance Sensing measure impedance in direct contact with the cell layer. These techniques, along with their integration and associated challenges, are reviewed by Ugodnikov et al.^[^ ^122^ ^]^ Integrating sensors could significantly enhance MPGFB studies by providing essential data for cell culture monitoring.

## 4 Engineering Behind the Microsystems

Microsystems integrating cells offer various advantages, with the most apparent one being the small scales that enable a reduced consumption of reactants and materials, sometimes of high cost. Another notable advantage is the potential for parallelization, facilitating high throughput screening of multiple conditions simultaneously. The versatility of microsystems is highlighted by their adaptability to diverse applications, spanning medicine, chemistry, physics, and more.

Additionally, due to their small scales, the Reynolds number of these microsystems is low, therefore allowing the exploitation of laminar flows, enhancing control over fluid dynamics. Furthermore, these microsystems offer the advantage of real‐time monitoring, a crucial aspect in understanding dynamic processes, such as cellular ones. Integrated electronics play a pivotal role in achieving this real‐time monitoring, allowing for precise control and observation of cellular behavior within the microenvironment.

In this third part, we focus on the engineering of the microsystems. We explore the technologies already employed in MPGFB and consider potential benefits from yet‐to‐be‐implemented technologies that could enhance the devices.

### 4.1 Materials and Microfabrication

This initial section will concentrate on the materials applicable in MPS. Some MPS incorporate a combination of materials to leverage the strengths of each and mitigate potential drawbacks. In this context, we highlight the materials utilized in MPGFB. It should be noted that the diversity of material used in MPGFB is very low, to our knowledge almost all devices are made from PDMS or Polymethylmethacrylate (PMMA). Therefore, we recommend readers to explore a comprehensive review written by Campbell et al. in 2021,^[^ ^123^ ^]^ which delves into materials beyond PDMS for broader organs‐on‐chip applications.

#### 4.1.1 MPS Scaffold

##### PDMS

The elastomer PDMS is the primary material utilized in microfluidic devices. Its transparency facilitates easy direct observation of the chip's interior using a simple optical microscope, despite a slight autofluorescence issue.^[^ ^124^ ^]^ PDMS is permeable to gas and biocompatible, making it convenient for cell culture. However, its high hydrophobicity, attributed to methyl groups on its surface, makes cell adhesion challenging without a coating. This hydrophobic property also leads its major limitation: it absorbs small hydrophobic molecules, thus impacting drug testing or marker measurements.^[^ ^125^ ^]^ PDMS is not compatible with most common organic solvents and will swell.

Despite these challenges, PDMS offers many advantages such as ease of fabrication, as well as cost‐effectiveness. However, it may not be the ideal choice for high‐throughput manufacturing. Before polymerization, PDMS is liquid and requires mixing with a cross‐linker, pouring into a mold, and curing in an oven for a few hours. The mold can be 3D printed or fabricated by lithography using photosensitive resin, like SU‐8 on a silica wafer. The final step typically involves adding a glass layer using plasma O_2_ activation for bounding. Moreover, PDMS's stiffness is tunable, ranging from very soft to relatively rigid, depending on the cross‐linker ratio during fabrication. Typically, the siloxane‐to‐cross‐linker ratio falls within the range of 9:1 to 14:1 for softer devices. It allows the building of dynamic structures, such as a stretching and compression movement,^[^ ^77^ ^]^ valves, and so on. However, its softness makes the integration of electronics difficult, even if a new generation of soft electronics has been emerging for the past decade.^[^ ^126^, ^127^ ^]^

PDMS is therefore very advantageous in the first steps of the MPS development and accounts for the vast majority of MPS, but is not sufficient for larger scales such as industrial ones.

##### Other Materials

PMMA is the 2nd most used material in MPS, beyond PDMS. It is a stiff, transparent, and biocompatible thermoplastic. Its fabrication process is less straightforward than PDMS and needs more techniques available such as milling, laser cutting, and hot embossing. However, it is very well suited for automatization and industrialization. Contrary to PDMS, it does not absorb small molecules.^[^ ^128^ ^]^ This makes it particularly suitable for applications involving drug screening or small molecule quantification. PMMA also offers excellent dimensional stability and is compatible with various surface treatments, but lacks the flexibility of PDMS, making it less suitable for dynamic components like valves or stretchable membranes. Therefore, PDMS is favored in laboratories for process development, whereas PMMA is mostly used when upscaling

MPGFB is so far made from either PDMS^[^ ^70^, ^71^, ^72^, ^75^, ^77^, ^81^, ^82^, ^83^, ^86^, ^87^, ^88^ ^]^ or PMMA.^[^ ^76^, ^84^ ^]^ Other types of materials can be used, glass, Cyclic Olefin Copolymer (COC), and SU‐8 being the most common ones, but have not been explored to our knowledge on the MPGFB field. Each of these materials has its own balance of benefits and limitations. For example, glass offers excellent chemical resistance and optical clarity but is brittle and expensive to process. COC is a promising thermoplastic due to its low autofluorescence and good chemical resistance, though its surface functionalization can be challenging due to its high hydrophobicity and chemically inert surface.^[^ ^129^ ^]^ One may also combine several of these material in order to combine the best of each (Glass & PDMS;^[^ ^86^ ^]^ Polycarbonate (PC)+PDMS;^[^ ^71^ ^]^ Stainless steel + Polytetrafluoroethylene (PFTE)).^[^ ^85^ ^]^

#### 4.1.2 Membrane Integration

Integrating membranes into microfluidic devices may be a challenging step. The thinner the membrane, the more difficult it is to handle, and the end‐device needs to be leak‐proof. The integrating method will depend on the material of the device and on the one of the membrane itself.

##### Ex Situ

The main method to place a membrane in a microfluidic chip is the “sandwich” method, where the membrane is placed between two PDMS parts. One convenient approach is using a PDMS membrane, as PDMS can be activated using oxygen plasma that will create oxygen radicals at its surface. Two activated parts of PDMS will be able to covalently bond together. However, PDMS membranes are thick and might not be the best choice physiologically speaking. Moreover, oxygen plasma is not necessarily compatible with eventual membrane functionalization and biological compounds. Chemical activation can be used, ′t Hart et al.^[^ ^72^ ^]^ used a salinization protocol on their PC membrane in order for it to bond to the activated PDMS device.

With stiffer outside materials, such as PMMA, PTFE, or stainless steel, screws can be used to seal the parts of the device together with a waterproof seal, making it easier to handle than PDMS.^[^ ^84^, ^85^ ^]^ Qu et al.^[^ ^71^ ^]^ used drilled PC to hold together the PDMS compartments and the porous membrane.

An innovative approach was explored by Mou et al.^[^ ^82^ ^]^ where they used electrospinning to create a thin silk fibroin membrane (3.5 µm). The membrane was then integrated into the PDMS device by using uncured PDMS as a glue.

##### In Situ

**Figure 8:**
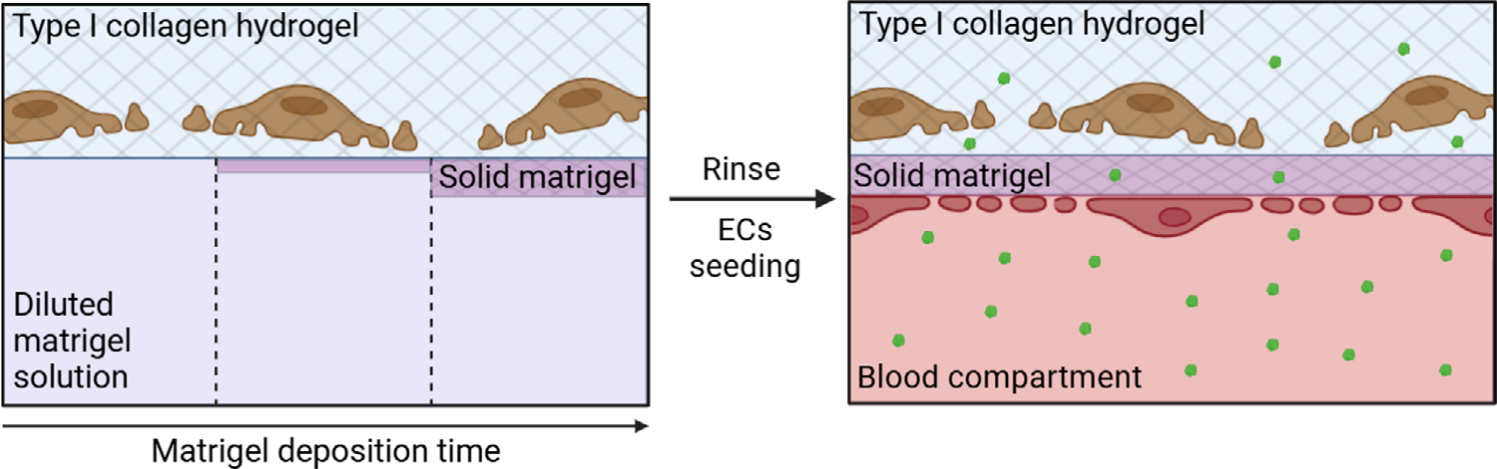
Illustration of the in situ fabrication process of Kim et al.^[^ ^88^ ^]^ On a type I collagen gel containing podocytes, a solution of diluted Matrigel is flown and becomes solid overtime. Once the membrane is formed, endothelial cells are deposited on the other side. (ECs = endothelial cells) The grid represents the solid parts of the device. Please note that all scheme are for understanding purposes and the relative sizes of the different components might not be representative of the real devices. Created in BioRender. Perry, G. (2025) https://BioRender.com/y90e964.

Building the GBM in situ is very challenging as well. Kim et al.^[^ ^88^ ^]^ managed to establish a protocol creating a thickness‐tunable GBM by varying the deposition time, achieving a really thin GBM (2.3 µm) (**Figure** **8**). However, a podocyte‐containing hydrogel was needed as a support for the GBM deposition, and the composition of the formed membrane was not controlled.

##### Other Techniques

3D bioprinting, where cells are embedded into a hydrogel and then printed into a desired shape, is a very convenient tool as it allows greater choice of shape. The hydrogel can be rather simple, such as collagen 1^[^ ^74^ ^]^ or using a decellularized matrix.^[^ ^80^ ^]^ However, this last option results in poor control over the composition and if animal matrix is used it might not be the most physiologically relevant method. Xie et al.^[^ ^73^ ^]^ used the properties of alginate to create a fiber‐shaped perfusable scaffold. Its thickness is still much greater than the in‐vivo GBM, as the glomerulus‐shaped nod thickness is 650 to 950 µm.

The microfabrication used in MPGFB remains quite simple, even if some innovative techniques have appeared the last few years. MPS remains a field mainly occupied by biologist, and would greatly benefit from collaborations which engineers specialized in microfabrication, or microelectronics to integrate micro sensors.

### 4.2 Commercial Devices

As MPS are more and more used in laboratories, companies have started commercializing ready‐to‐use devices. While transwells are commonly used for cell study, and glomerular cells are no exception.^[^ ^86^, ^87^, ^89^ ^]^ However, it is commonly admitted that transwells do not reproduce a physiological relevant support, and flow is difficult to integrate. In MPGFB, Organoplate from Mimetas^[^ ^78^, ^79^, ^110^ ^]^ and Emulate chips^[^ ^69^, ^81^ ^]^ are used.

Commercial devices are convenient, offering high throughput, low batch to batch variation, and accessories such as media carrier, gravity‐driven flow, integrated TEER measurement, and so on. However, they remain expensive and might not be adapted for all cultures. For example, the membrane in Emulate S1 is 50 µm thick with pores of 7 µm diameter, far from the in‐vivo GBM.

The last 10 years, a lot of companies specializing in organ‐on‐chip appeared. Even if they are not used in MPGFB to our knowledge, some of their technology could be applied to MP‐barriers: NeoBento from Netri, Predict96 from Draper, Alveolix, and AIM Biotech are among them.

## 5 Applications

Kidneys play a crucial role in filtering and clearing drugs from the body, making them particularly susceptible to drug‐induced toxicity. This chapter delves into the mechanisms by which various nephrotoxic drugs, such as aminoglycosides, Adriamycin, and NSAIDs, impact renal function, potentially leading to acute kidney injury (AKI). We will also focus on diseases that might lead to CKD, such as diabetes, hypertension, genetic, or autoimmune diseases. This section highlights recent advances in MPGFB for drug testing and disease modeling, underscoring the potential of these innovative platforms to transform our understanding of kidney‐related diseases and drug toxicities.

### 5.1 Drug Testing

Kidneys, because of their drug‐clearance role and the high drug concentrations reach in the tubules, are particularly vulnerable to drug‐induced toxicity. They can have different impacts on the kidneys, sometimes provoking AKI. Here are listed some common nephrotoxic drugs and their effect.

Adriamycin, or doxorubicin, is a chemotherapeutic drug. It is a commonly used drug for assessing the reaction of kidney models to drug injury.^[^ ^63^, ^71^, ^80^, ^81^ ^]^ It was reported that in response to Adriamycin, glomerular cells decrease their production of specific proteins, resulting in drastic thinning of the glomerular endothelial glycocalyx, and therefore proteinuria, underscoring its importance in the filtration barrier.^[^ ^63^ ^]^

NSAIDs are commonly used world‐wide against fevers and as painkillers. However, they act by suppressing the synthesis of prostaglandin, resulting in the impossibility of the afferent arteriole to dilate and therefore regulate the blood flow. This can be particularly concerning in the older population and for patients with existing diseases as it can lead to kidney failure.^[^ ^130^, ^131^ ^]^

Aminoglycosides are antibiotic medications used against gram‐negative bacteria by disturbing the production of certain proteins and permeability their membrane.^[^ ^132^ ^]^ The molecule is excreted through the urine, but a small portion is retained, especially in the epithelial cells of the proximal tubule where they accumulate,^[^ ^133^ ^]^ inducing changes and eventually rupture of lysosomes, therefore provoking tubular dysfunction.^[^ ^134^, ^135^ ^]^

#### In MPGFB

Decades are required to develop a new drug. More than 80% of drugs will fail in clinical trials, inducing high developmental cost. MPS offers a promising and more efficient alternative to animal testing. In December 2022, the FDA Modernization Act 2.0 was approved, allowing new drugs to go into clinical trials without the need of animal experiments. Since then, a few potential drugs tested on MPS were allowed into clinical trials.^[^ ^136^, ^137^ ^]^ A recent review of the advances of MPS regarding drug testing has recently been published.^[^ ^138^ ^]^

As of today, MPGFB alone has not been used to pass a drug into clinical trial. However, drugs such as Adriamycin is often used in MPGFB to demonstrate the potential use of these devices for medical screening.^[^ ^71^, ^73^, ^77^, ^80^, ^81^, ^82^, ^85^, ^86^ ^]^ Those articles reported cell death, albumin leakage, foot processes effacement, reorganization of actin filaments, and decrease expression of functional markers. Cisplatin, another anticancer drug, and Puromycin Aminoglucoside (PAN), are also used even if their toxicity is directed to the proximal tubule, making this choice questionable for studying glomerular cells.^[^ ^71^, ^76^, ^85^ ^]^ We also note the absence of anti‐VEGF drugs (e.g., bevacizumab) that decorrelate endothelial cells from podocytes.

Not all studies delve into the mechanisms of toxicity, and the associated markers. It is worth noting that the range of concentration varies from two to three orders of magnitude, and the exposure time ranges from 1 to 7 days (**Table** **6**). This variation hinders both the comparison between articles and the goal of standardization.

**Table 6:** List of the drugs and their concentration used to induce glomerular injury. Unless stated otherwise, the drug carrier is an aqueous media, either cell culture media, cell differentiation media, PBS, or one of the latter slightly modified.

Drug	Ref	Concentration [µg·mL^−1^]	Exposure time [days]	Conclusion
Cisplatin	[76]	5 – 10 – 15	1.5	Cell death, recovery after 96 h.
	[71]	10	2	Impact limited on podocytes.
	[85]	1.9–19 in DMSO 1%	7	Impact limited on podocytes. Increase of glucose clearance.
PAN	[86]	0.3 → 221	3	Estimation of LD_25/50/75_. No significant difference between static and dynamic conditions.
	[88]	100	1	Increase in permeability. Reduction of cell markers.
	[87]	5–15–29	1	Increase in permeability. Downregulation of cell markers. Protective effect of dexamethasone
	[79]	10	5	Loss of actin, compromised permselectivity
Doxorubicin / Adriamycin	[86]	0.005 → 54	1	Estimation of LD_25/50/75_. Difference between static and dynamic conditions for LD_50_ and LD_75_
	[82]	0.5 in DMSO 0.1%	3	Altered expression of podocin, foot processes loss, compromised selectivity
	[77]	0.25–0.5–1	5	Podocyte detachment, compromised permselectivity
	[71]	1.25	2	Podocyte death, foot processes disappearance.
	[81]	0.5	2	Cell detachment, compromised permselectivity and actin reorganization
	[80]	0.5–1	3	Cell death, reduced markers, compromised permselectivity.
	[73]	1	2	Compromised permselectivity
	[85]	0.25–2.5	7	Cell detachment

Doi et al.^[^ ^86^ ^]^ was able to determine the LD_25_ (Lethal Dose 25%), LD_50_, and LD_75_ of Adriamycin and PAN, and compare them in static versus dynamic conditions. Significant differences were observed in some cases, and their value of Adriamycin LD_50_ was reported to be lower than the one calculated on mouse model. PAN was also used by Korolj et al.,^[^ ^87^ ^]^ where they observed its toxicity on podocytes, but also successfully looked into the protective effect of dexamethasone.

Kim et al.^[^ ^88^ ^]^ looked into the influence of VEGF‐A, known to be overproduced by podocytes in nephrotic syndrome, on GEnCs. They observed an increase in permeability and decrease in tight junction marker. This finding is especially interesting, as VEGF‐A serum levels are not correlated with urinary levels, making it hard to study in vivo.

As demonstrated in this paragraph, MPGFB are promising tools to study drug toxicity in the context of clinical trials. However, before reaching that step, more studies must be performed, especially comparing in vivo toxicity levels with the one obtained through MPS studies.

#### 5.1.1 Disease Modeling

##### 5.1.1.1 Diabetic Nephropathy

Diabetes affects more than 500 million people, type 1 (10%) and type 2 (90%) included, in the world and kills approximately 2 million each year.^[^ ^139^ ^]^ Around 40% of these people are or will be affected by diabetic nephropathy (DN).^[^ ^59^ ^]^ Diabetes is responsible for almost half the CKD in the US.^[^ ^140^ ^]^ DN is classically characterized by increased protein excretion in urine and low glomerular filtration rate.^[^ ^141^ ^]^ It is the result of several physiological causes, such as the increase of glomerular volume and the thickening of the GBM. Whereas endothelial glycocalyx thins, endothelial fenestration diminishes and podocytes detach in the latest stages of the disease.^[^ ^142^, ^143^ ^]^ The main reasons for these changes are the hyperglycemia and the hypertension often accompanying diabetes. The metabolic pathways are complex and nicely described in these articles.^[^ ^144^, ^145^, ^146^ ^]^

##### In MPGFB

Normal fasting blood sugar ranges around 4–6 mM, and levels above 15 mM usually require immediate medical attention. MPGFB have been used to study the effect of glucose on glomerular cells. High glucose level induces increased permeability.^[^ ^70^, ^79^, ^80^ ^]^ More interestingly, Singh et al.^[^ ^80^ ^]^ observed the increase of ICAM‐1, an inflammatory marker, decrease of functional markers, and suppression of the tight function protein ZO‐1. The reduction of ZO‐1 was also observed by Wang et al.^[^ ^70^ ^]^ Moreover, they showed radical oxygen species production with high glucose, and increase of GLUT‐1, a specific protein responsible, amongst other, for glucose transport into cells, and insulin‐stimulated glucose uptake. This work demonstrated that increased GLUT‐1 could be potentially used for diabetic nephropathy detection. However, diabetes is a systemic disease whose effect goes beyond the kidney, and is more complex than high blood sugar. Therefore, it would be more correct to say that these devices study the effect of high blood sugar on cells rather than diabetic nephropathy.

#### 5.1.2 Hypertension and Cardiovascular Diseases

The relation between hypertension and renal damage is complex. Since the 1920′s it has been strongly suspected that kidney damage induces high blood pressure, increasing the risk of cardiovascular diseases (CVD). It was later discovered that the link between both was less straightforward, as patients with hypertensive family background were more at risk of developing CKD.^[^ ^60^, ^61^ ^]^ More recently, it has been shown in patients with proteinuria that low blood pressure slows the progression of renal diseases.^[^ ^147^ ^]^ Therefore, CKD can be a cause as well as a consequence of hypertension. The complex pathophysiological aspects are described by Singh.^[^ ^148^ ^]^ Moreover, patients with CKD are more at risk of dying from CVD than needing dialysis, illustrating the tragic relationship between both pathologies.^[^ ^149^ ^]^

It is worth noting that diabetes, hypertension, and CKD are often intricately linked, as they have several predisposition factors in common such as male gender, high body mass index, smoking, and proteinuria. They can also facilitate the apparition of one another.^[^ ^150^ ^]^

##### In MPGFB

A few MPGFB declare to study the effects of hypertension on glomerular cells. However, hypertension is complex and linked to vasoconstriction and other markers. Those studies mostly investigate the effect of high shear stress rather than hypertension. There are several methods used to apply those high shear stresses. A straightforward approach is to directly apply pressure through a pressure pump. It is easier to implement in transwells, and the pressures studied goes from 10 to 4700 Pa.^[^ ^86^, ^89^ ^]^ Normal blood pressure is in the range of 10kPa,^[^ ^151^ ^]^ making the studied values far from physiological reality, due to the devices. Dai et al.^[^ ^76^ ^]^ varied the length of the outlet channel, modeling the arteriole, which increase the hydraulic resistance and, therefore the pressure. They showed that with increase pressure, more fluid would pass the barrier, but did not see a change in albumin retention. Lastly, Zhou et al.^[^ ^75^ ^]^ choose to increase the flow rate, which increases the shear rate and damages the cells, but not necessarily increases the pressure inside the channels. The injury marker vWF was increased, and they reported the reorganization of actin filaments.

Overall, even if the negative impact of high shear stress on the cells has been studied on MPGFB, the extend of the studies remain limited with little study of markers and morphology. However, such devices cannot pretend to reproduce the conditions of hypertension.

#### 5.1.3 Genetic Diseases

Genetic mutations can lead to a variety of kidney diseases, each with unique clinical manifestations and severity. These inherited conditions highlight the critical role of specific genes in kidney function and overall health. Some examples will be given. Pierson Syndrome affects the β2 strand of laminin. It usually leads to end‐stage renal failure, and death without dialysis, in the first weeks or months of life.^[^ ^152^ ^]^ Hereditary Angiopathy with Nephropathy, Aneurysms, and muscle Cramps Syndrome affects the COL4A1 gene coding for the α1(IV) chain on chromosome 13.^[^ ^153^ ^]^ Symptoms can vary significantly among affected individuals and may include porencephaly, nephropathy, retinal hemorrhages, vision loss, and muscle cramps.^[^ ^154^ ^]^ However, the only syndrome studied using MPGFB is AS. It was first identified in 1927 by Cecil Alport and affects approximately 1 in 50 000 people.^[^ ^155^ ^]^ It arises from an inherited flaw in the genetic code responsible for type IV collagen, thereby impacting all parts of the body where type IV collagen is found such as ears, eyes, and kidney. Different forms of the syndrome exist, each with distinct symptoms and varying severity. The initial gene mutation identified was COL4A5,^[^ ^156^ ^]^ coding for the chain a5(IV) and situated on the X chromosome, explaining why man where commonly more affected than woman, as they only have a single X chromosome.^[^ ^157^ ^]^ It was later found that the genes COL4A3 and COL4A4 were also mutated.^[^ ^158^ ^]^ The more severe forms can cause kidney failure and deafness in the early adolescence, and in some cases, death.

These syndromes highlight how a single genetic mutation can have profound and often life‐threatening effects. MPS are very promising tools for the study of genetic diseases by using disease‐carrying stem cells.

##### In MPGFB

Petrosyan et al.^[^ ^79^ ^]^ conducted an initial study using podocytes derived from the amniotic fluid of patients with AS. They observed that chips created with these podocytes exhibited significantly higher albumin leakage, demonstrating the potential of iPSCs in MPGFB to study genetic diseases using cells from affected patients.

#### 5.1.4 Autoimmune Diseases

Auto‐immune diseases represent a significant category of kidney pathology, where the immune system mistakenly attacks the body's own tissues, including the kidneys. These conditions can lead to severe and rapidly progressing kidney damage. Lupus nephritis is one of the most severe organ manifestations of Systemic Lupus Erythematosus, a serious systemic autoimmune disease with unknown causes. The incidence is really depending on the ethnicity and varies from 4 per 100 000 people per year to 10.65 per 100 000 people per year. The autoimmune mechanisms are complex and can be more pathogenic in case of genetic factors.^[^ ^159^ ^]^ IgA nephropathy, also known as Berger's disease, is a common cause of glomerulonephritis, whose incidence is around 31 to 45 per million population per year depending on the studied population.^[^ ^160^ ^]^ Its characteristic feature is the poorly O‐glycosylated Immunoglobulin A mesangial deposits. Various factors (genetic and environmental) are driving the disease's mechanisms. Its progression is hypothesized to follow a four‐hit sequence.^[^ ^161^, ^162^ ^]^ Membranous glomerulonephritis (MG), also known as membranous nephropathy (MN), accounts for approximately 30% of all nephrotic syndromes in the adult population and, though rare, can also affect children.^[^ ^163^ ^]^ It affects around 1 in 100 000 people and can be associated with medications or other conditions, such as Lupus. MG is primarily characterized by thickening of the glomerular capillary wall due to the accumulation of immune deposits, resulting in proteinuria and, at times, hematuria. The outcomes can vary significantly, ranging from acute kidney failure to spontaneous remission in nearly one‐third of cases. MG has undergone extensive study and review; interested readers are directed to these references^[^ ^164^, ^165^ ^]^ for further information. Goodpasture's Syndrome is a rare syndrome, occurring in less than one in a million cases.^[^ ^166^ ^]^ It is characterized by the attack of antibodies and T‐cells on the NC1 domain of the α3 chain.^[^ ^167^ ^]^ Patients typically require treatment involving plasma exchanges and immunosuppressants.^[^ ^168^ ^]^

##### In MPGFB

Petrosyan et al.^[^ ^79^ ^]^ also investigated MN, a condition characterized by anti‐podocyte antibodies in the sera of affected individuals. When serum from MN patients was used in MPGFB, it induced albumin leakage, in contrast to serum from patients with non‐autoimmune conditions such as AS, polycystic kidney disease, and focal segmental glomerulosclerosis. The study focused on PLA2R, the primary podocyte target antigen, confirming the correlation between PLA2R expression and the podocytes' response to MN serum. It also underscored the importance of using relevant models, as chips made with immortalized podocytes did not respond to MN serum, unlike those made with primary podocytes or podocytes derived from amniotic fluid. Furthermore, the beneficial effect of α‐Melanocortin stimulating hormone, which mimics a treatment used in MN, was successfully replicated in MPGFB. This group further advanced the analysis of MN in a study led by Zhang et al.,^[^ ^78^ ^]^ utilizing both MPGFB and in vivo mouse models. They demonstrated that C3a/C3aR signaling plays a critical role in the complement‐mediated pathogenesis of MN, identifying it as a potential therapeutic target for the condition. More recently, Kim et al.^[^ ^169^ ^]^ used a customized microfluidic device in which they inserted a transwell covered by glomerular cells on each side. Only podocyte are exposed to a bi‐directional flow generated by a perfusion rocker. In exposing the glomerular endothelial cells to 0.5% serum from patients suffering from IgA nephropathy, MN, minimal change disease, and lupus nephritis (five patients for each disease), they assessed podocyte viability and the albumin permeability after 24 h. Their results showed significant reduction in podocyte viability for the majority of the conditions, while the albumin permeability was significantly increased for three MN's sera and one IgA nephropathy's serum. Nevertheless, the readouts of their experiments and the physiological relevance of their device are limited.

## 6 Conclusion and Perspectives

### 6.1 Need of Standardization and Validation

MPGFB are still in their premises, the first one having been developed in 2016.^[^ ^75^ ^]^ Developing a new technology takes times, and challenges must be tackled. The two main challenges that MPS will face in the next few years are the need for standardization and validation. Throughout this review, we have seen the disparities between major parameters such as flow, seeding densities, geometries, materials used, and so on. This is a major hindrance to standardization, and as a consequence to MPS being used globally for any application.

Moreover, MPS in general lack validation: there is not enough proof on whether they are reliable models when it comes to modeling human physiology. Validation is difficult due to the lack of data regarding human physiology. The kidney is especially difficult to image due to its small size (≈200 µm diameter^[^ ^170^ ^]^) and his high vascularization. Its study is often indirect, using urine or blood tests.^[^ ^171^ ^]^ Therefore, one idea put forward by some would be to use the data available on animals already accumulated by years of animal experiment, to validate the devices with animal cells. Then, if MPS using animal cells are reliable tools to replicate animal physiology, it would be a great hint that using human cells may provide reliable results.

Additionally, comparisons between MPS data and outcomes from existing in vivo models, such as genetically modified animals or other disease models, could strengthen the validation process. Studies correlating MPS results with clinical observations or patient‐derived data, when available, could also help bridge the gap. Currently, most studies validate their platforms by replicating well‐documented pathological effects, such as Adriamycin‐induced toxicity or the consequences of high shear stress. While informative, these validations often remain superficial and lack mechanistic depth. Qualitative observations, though useful, are not sufficient for rigorous model evaluation. Where possible, the incorporation of quantitative endpoints, such as permeability coefficients, LD₅₀ values, or molecular transport rates, is essential to reinforce both reliability and translatability.

However, such validation efforts cannot be dissociated from the need of standardization. Without common protocols, reference materials, and reproducibility criteria, it remains difficult to compare results across studies or to move toward regulatory acceptance. Achieving consensus on critical design and reporting parameters is thus a prerequisite for the integration of MPS technologies, including MPGFB, into drug development pipelines or precision medicine strategies.

### 6.2 Setting Clear Limits to MPGFB Relevance

Studying systemic diseases such as diabetes or hypertension using MPGFB holds limited interest, as accurately replicating in vivo conditions, such as hormonal and immune influences, remains highly challenging. Instead, we believe these devices could have a significant impact on advancing research into drug toxicity, genetic disorders, and autoimmune diseases. An overlooked disease is the idiopathic nephrotic syndrome, where the primary cause of disease is unknown but a circulating factor in the serum is suspected to be the main cause. So far, there is just one article that tested this hypothesis in MPGFB for minimal change disease.^[^ ^169^ ^]^ Albumin permeability results were not significant after 24 h exposure suggesting that longer exposition is required or that other physiological parameters are missing such as perfusion on the endothelial compartment, mesangial or immune cells for example.

Moreover, while in this review we insisted on the limits of those devices, the similarities must not be undermined. In systems where the glomerular basement membrane is produced endogenously by co‐cultured endothelial cells and podocytes, MPGFB provide a unique opportunity to study GBM composition, secretion dynamics, and remodeling in both physiological and pathological contexts. These systems also allow for the investigation of cellular cross‐talk between the glomerular endothelial cells, podocytes, and mesangial cells when integrated, which is crucial for maintaining the integrity and function of the filtration barrier. By studying the expression of specific cell surface markers, it is possible to gain deeper insights into how these cell types communicate and coordinate their functions within the glomerulus. Understanding these interactions and markers is critical for uncovering the molecular mechanisms of glomerular diseases, where cell signaling and marker expression are often altered.

### 6.3 Future Directions

The next step for MPS is often considered as the “body‐on‐chip” where all of the organs are connected and interact with each other. However, this ambitious goal cannot be achieved without the development of the organ as a first step. As we saw the kidney is a complex entity, and one cannot pretend achieving a “kidney‐on‐chip” when only focusing on one part such as the tubule or the glomerulus. MPGFB are not yet mature in vitro models of the GFB, let alone of the glomerulus, which is a far more complex entity, involving more proteins, cell types, and geometrical complexity. Therefore, achieving a reliable glomerulus‐on‐chip, and then kidney‐on‐chip, must be the next goal. This requires significant advances in the engineering of MPGFB, particularly in replicating the glomerular basement membrane. Existing devices mostly feature GBMs that are too thick and composed of non‐physiological materials, inevitably compromising the physiological relevance and reliability of the models. Techniques such as electrospinning or in‐situ membrane generation seem promising but have not yet reached complete physiological relevance. Moreover, none of the MPGFB uses sensors, which could be a give valuable insight on parameters such as O_2_ concentration, or membrane permeability. The electrical forces that drive the glomerular filtration are not perfectly understood, and using electrical sensors in those devices could greatly help our understanding of these mechanisms.

Finally, the lifespan of MPGFB remains relatively short, typically lasting no more than two weeks under optimal conditions. One of the main limitations is the progressive dedifferentiation of podocytes, a critical issue that compromises the long‐term stability and functionality of the glomerular filtration barrier. Although some research groups^[^ ^73^, ^79^, ^99^ ^]^ state that their MPGFB can remain functional for several weeks after barrier formation, there is currently no in‐depth study systematically evaluating this aspect. Continuous monitoring of barrier permeability, for instance through TEER measurements, could provide valuable insights into the structural and phenotypic integrity of glomerular cells over time. An increase in permeability may reflect early signs of podocyte dedifferentiation, which can manifest through morphological changes such as foot process effacement or loss of fenestrations. As highlighted by May et al.^[^ ^172^ ^]^ podocyte dedifferentiation shares key features with podocytopathies, reinforcing the need for more assessments of MPGFB models.

To conclude, the results observed in this review are the promise of a revolution in bioengineering that will not only affect drug discovery, but also our understanding of diseases and personalized medicine.

## Conflict of Interest

The authors declare no conflict of interest.

## Supporting information



Supporting Information
