# Supplementary material for: Microphysiological Glomerular Filtration Barriers: Current Insights, Innovations, and Future Applications
Source: Adv Biol (Weinh). 2025 Jul 7;9(9):e00108. doi: 10.1002/adbi.202500108 (PMC12447127; doi:10.1002/adbi.202500108)
Supplement: Supplementary file 1 — Supporting Information [file ADBI-9-e00108-s001.docx]

**Supporting Informations**

**Microphysiological Glomerular Filtration Barriers: Current Insights, Innovations, and Future Applications**

*Manon Miran^1,2,3^, Kieu Ngo^2^, David Buob^1^, Hanna Debiec^1^, Pierre Ronco^1^, Guillaume Perry^1,3*^*

**Affiliations:**

^1^ Sorbonne Université, Inserm, Common and Rare Kidney Diseases: from Molecular Events to Precision Medicine, CoRaKiD, F-75020 Paris, France

^2^ Sorbonne Université, CNRS, Laboratoire de Réactivité des Surfaces, LRS, F-75005 Paris, France

^3^ Sorbonne Université, CNRS, Université Paris Saclay, CentraleSupelec, Laboratoire de Génie Electrique et Electronique de Paris, GeePs, F-75005, Paris, France

*Corresponding author, Guillaume.perry@sorbonne-universite.fr

Methodology used to generate the Figure 3.

Data were extracted from Pubmed on January 17^th^, 2025 according to the following instructions:

- Kidney data: Kidney-on-a-chip OR Kidney-on-chip OR Kidney on a chip OR Kidney on chip OR Glomerulus-on-a-chip OR Glomerulus-on-chip OR Glomerulus on a chip OR Glomerulus on chip OR Tubule-on-a-chip OR Tubule-on-chip OR Tubule on a chip or Tubule on chip

This search provided 1553 results.

- Glomerulus data: Glomerulus-on-a-chip or Glomerulus-on-chip or Glomerulus on a chip or Glomerulus on chip

This search provided 105 results.

- Tubules data: Tubule-on-a-chip or Tubule-on-chip or Tubule on a chip or Tubule on chip

This search provided 279 results. No distinction has been made between proximal and distal tubules.

In the 3 cases, results were then checked manually to insure their relevance and were classified in 4 different categories or in 5 for the kidney data as follow:

1. Research articles
2. Review articles
3. Others: Editorial, commentaries, forum
4. Out of the scope (not relevant regarding the scope of the search and article’s correction)
5. Organoïds articles (for the kidney data)

| Auteur | Shear stress  [dyn.cm^-2^] | Flow rate  [µL.min^-1^] | Equipment used |
| --- | --- | --- | --- |
| Dai *et al.*(2023) ^[76]^ | 0.009 | 3.3 | Peristaltic pump |
| Doi *et al.*(2023) ^[86]^ | N.D. | 0.07 0.17 0.35 | Syringe pump for medium supply  Air pressure generator for streching |
| Fallon *et al.*(2023) ^[84]^ | 0.031  0.015  0.0077  0.0025 | 100 50 25 8 | Cole-parmer peristaltic pump |
| Mou *et al.*(2024) ^[82]^ | N.D. | 1 | Peristaltic pump |
| Musah *et al.*(2017) ^[77]^ | 0.0007 (urinary) 0.017 (capillary) | 1 | Peristaltic pump |
| Pajoumshariati *et al.*(2023) ^[69]^ | 0.0007 (urinary) 0.136 (capillary) | 1 | Gravity driven flow (ZOE emulate) |
| Petrosyan *et al.*(2019) ^[79]^ | 0.117 | N.D. | Gravity-driven flow (Mimetas)  7° rock every 10min |
| Qu *et al.*(2018) ^[71]^ | N.D. | 1 | Peristaltic pump |
| Roye *et al.*(2021) ^[81]^ | 0.0007 (urinary) 0.017 (capillary) | 1 | Gravity-driven flow (Emulate Pod) |
| Schmieder *et al.*(2019) ^[89]^ | N.D. | 60 | Pressure regulated |
| Singh *et al.*(2023) ^[80]^ | N.D. | 0.1-10 | Peristaltic pump |
| Wang *et al.*(2017) ^[70]^ | N.D. | 2.5 | N.D. |
| Xie *et al.*(2020) ^[73]^ | 3 to 9 | 14 to 42* | Gravity-driven flow  45° rock |
| Zhang *et al.*(2024) ^[78]^ | 0.117 | N.D. | Gravity-driven flow  7° rock every 10min |
| Zhou *et al.*(2016) ^[75]^ | 0.001  0.002  0.003 | 5  10  15 | Syringe pump |

Table S1: Flow rates and shear stresses used in MPGFB. *calculated with data of the article (N.D. = Not Disclosed).

| **Auteur** | **Cell surface density [cells.cm^-2^]** | **Cell initial concentration [cells.mL^-1^]** | **Total number of cells** |
| --- | --- | --- | --- |
| Dai *et al.*(2023) ^[76]^ | N.D. | Podo: 1E7  Endo: 2E5 | Podo: 3E5 Endo: 2E5 |
| Doi *et al.*(2023) ^[86]^ | Podo: 1.5E5 | N.D. | N.D. |
| Fallon *et al.*(2023) ^[84]^ | Podo: 1.6E5* Endo: 6.7E5* | N.D. | Podo: 1.5E5 Endo: 4E5 |
| Flegeau *et al.* (2019) ^[74]^ | N.D. | Gel Endo: 3.5E7  Podo: 5E4 | Podo: 5E4 |
| Kim *et al.* (2023) ^[88]^ | N.D. | Gel Podo: 1E6  Endo: 0.5 - 1 - 2 E6 | Endo: 2.5E4 - 5E4 - 1E5 |
| Korolj *et al.* (2018) ^[87]^ | Podo: 1.5E4 or 5E4 | N.D. | N.D. |
| Mou *et al.*(2024) ^[82]^ | Podo: 8.3E3* Endo: 3.5E5* | Podo: 2E5  Endo 6E6 | Podo: 6E3 Endo:1.5E5 |
| Musah *et al.*(2017) ^[77]^ | (If 18mm length) Podo: 5.6E4* Endo: 1E5* | N.D. | Podo: 4E4 Endo: 4E4 |
| Pajoumshariati *et al.*(2023) ^[69]^ | Endo: 1.3E5* Podo: 1.5E4* | N.D. | Endo: 8.5E4  Podo: 1.7E4 Mesangial: 2.2E5 cells in gel |
| Petrosyan *et al.*(2019) ^[79]^ | Podo & Endo: 1.9E5* | Podo & Endo: 1.5E7 | Podo & Endo: 3E4 |
| Qu *et al.*(2018) ^[71]^ | Podo: 6E4 Endo: 1E5 | N.D. | N.D. |
| Roye *et al.*(2021) ^[81]^ | Podo: 7E4* Endo: 1.3E5* | N.D. | Podo: 8E4 Endo: 9E4 |
| Schmieder *et al.*(2019) ^[89]^ | N.D. | N.D. | N.D. |
| Singh *et al.*(2023) ^[80]^ | N.D. | Gel podo: 2E7  Gel endo: 1E7 | N.D. |
| ‘t Hart *et al.* (2023) ^[72]^ | N.D. | Podo: 1E6  Endo: 2E7 | N.D. |
| Tabuchi *et al.* (2021) ^[83]^ | N.D. | Podo: N.D.  Endo: 1E7 | N.D. |
| Wang *et al.*(2017) ^[70]^ | N.D. | 1E4 glomeruli per ml | N.D. |
| Xie *et al.*(2020) ^[73]^ | Endo: 2.5E6* | Podo: 4E6  Endo: 3E7 | Podo: 1.2E5 Endo: 1.2E5 |
| Zhang *et al.*(2024) ^[78]^ | Podo & Endo: 1.9E5* | Podo & Endo: 1.5E7 | Podo & Endo: 3E4 |
| Zhang and Mahler (2023) ^[85]^ | Podo: 1E5 Endo: 1E5 | N.D. | N.D. |
| Zhou *et al.*(2016) ^[75]^ | N.D. | N.D. | N.D. |

Table S2: Seeding densities used in MPGFB. *Calculated with the data of the article, the surface used was the whole surface of the channels, rather than just the surface of the membrane (N.D. = Not Disclosed).
